# Recent advances, challenges and updates on the development of therapeutics for malaria

**DOI:** 10.17179/excli2023-6856

**Published:** 2024-05-06

**Authors:** Rimmy Nandal, Davinder Kumar, Navidha Aggarwal, Virender Kumar, Balasubramanian Narasimhan, Rakesh Kumar Marwaha, Prabodh Chander Sharma, Surender Kumar, Nitin Bansal, Hitesh Chopra, Aakash Deep

**Affiliations:** 1 Shri Baba MastNath Institute of Pharmaceutical Sciences and Research, Baba Mast Nath University, Asthal Bohar, Rohtak-124001, Haryana, India; 2 College of Pharmacy, PGIMS University of Health Sciences, Rohtak-124001, Haryana, India; 3 MM College of Pharmacy, Maharishi Markandeshwar (Deemed to be University), Mullana, Ambala 133207, India; 4 Department of Pharmaceutical Sciences, Maharishi Dayanand University, Rohtak 124001 Haryana, India; 5 Department of Pharmaceutical Chemistry, School of Pharmaceutical Sciences, Delhi Pharmaceutical Sciences and Research University, New Delhi 110017, India; 6 Department of Chemistry, Chaudhary Bansi Lal University, Bhiwani-127021, India; 7 Department of Pharmaceutical Sciences, Chaudhary Bansi Lal University, Bhiwani-127021, Haryana, India; 8 Department of Biosciences, Saveetha School of Engineering, Saveetha Institute of Medical and Technical Sciences, Chennai - 602105, Tamil Nadu, India

**Keywords:** antimalarials, clinical trials, malaria, tafenoquine, cipargamin, artefenomel

## Abstract

Malaria has developed as a serious worldwide health issue as a result of the introduction of resistant *Plasmodium* species strains. Because of the common chemo resistance to most of the existing drugs on the market, it poses a severe health problem and significant obstacles in drug research. Malaria treatment has evolved during the last two decades in response to *Plasmodium falciparum* drug sensitivity and a return of the disease in tropical areas. *Plasmodium falciparum* is now highly resistant to the majority of antimalarial drugs. The parasite resistance drew focus to developing novel antimalarials to combat parasite resistance. The requirement for many novel antimalarial drugs in the future year necessitates adopting various drug development methodologies. Different innovative strategies for discovering antimalarial drugs are now being examined here. This review is primarily concerned with the description of newly synthesized antimalarial compounds, i.e. Tafenoquine, Cipargamin, Ferroquine, Artefenomel, DSM265, MMV390048 designed to improve the activity of pure antimalarial enantiomers. In this review, we selected the representative malarial drugs in clinical trials, classified them with detailed targets according to their action, discussed the relationship within the human trials, and generated a summative discussion with prospective expectations.

## Introduction

Although recent developments give cause for hope, malaria infections and deaths are still at an unacceptable level and have returned in numerous contexts (Jagannathan and Kakuru, 2022[48]). Malaria is a significant public health issue (Nandal et al., 2019[75]) and is viewed as the evolutionary arms race between the host and parasite population (Band et al., 2022[11]) due to its high yearly death and morbidity (Egwu et al., 2022[35]). Malaria is a life-threatening, mosquito-borne disease caused by *plasmodium *parasites (Wu et al., 2022[118]). Malaria is one of the most deadly infectious diseases in humans. It is clinically and economically troublesome because it is prevalent in impoverished countries and areas, severely impeding socioeconomic progress. These parasites infect humans and other animals, including reptiles, birds, and mammals. Over 200 *Plasmodium *species have been formally described, with each species infecting a specific range of hosts. Only five Plasmodium species commonly infect humans and cause malaria in broad parts of the world: *P. falciparum*, *P. vivax*, *P. malariae*, *P. ovale* and *P. knowlesi*. The first four are unique to humans, although *P. knowlesi* is usually found in macaque monkeys and causes zoonotic malaria widely in South East Asia (Sato, 2021[96]; Uneke, 2007[110]). Malaria has always returned like a "phoenix from the ashes" despite numerous human attempts to stop its growth and spread (Bhattacharjee and Shivaprakash, 2016[13]; Mukherjee et al., 2008[72]). Before 2010, roughly half of the world's population (3.3 billion people) was at risk of malaria infection, with 300 to 500 million cases reported annually, resulting in approximately 1.24 million fatalities. The WHO reported an 85 percent decline in malaria cases in 34 malaria-eliminating countries, from 1.5 million in 2000 to 232,000 in 2010. From 2004 to 2010, malaria deaths fell from 1.82 million to 1.24 million (Cohen et al., 2012[22]). Globally, an estimated 219 million malaria cases occurred in 2017, compared to 239 million in 2010 and 217 million in 2016.

Malaria morbidity reductions from 2015 to 2017 were not considerable, even though there were an estimated 20 million fewer malaria cases in 2017 than in 2010. Malaria killed an estimated 405,000 people worldwide in 2018, with 228 million cases. In 2019, there were an estimated 229 million malaria cases in 87 malaria-endemic countries, with a 9 million malaria case drop. The World Malaria Report, released in December 2021, represents the unique difficulties confronting the global malaria community. The analysis demonstrated the catastrophic toll of malaria, with an anticipated 627,000 people death in 2020 (Monroe et al., 2022[70]). Globally, 247 million malaria cases were anticipated in 2021 in 84 malaria-endemic countries, up from 245 million in 2020. Malaria case incidence (cases per 1000 persons at risk) fell from 82 in 2000 to 57 in 2019 but increased to 59 in 2020. There was no difference in case occurrence between 2020 and 2021. The 2020 surge was linked to service disruptions caused by the covid-19 pandemic (World Health Organization). Malaria is characterized by repeated chills, a high fever, nausea, and vomiting and is spread by mosquito bites (Deshpande and Kuppast, 2016[29]). In the malaria-affected population, cerebral malaria is the leading cause of death. The mortality survival rate of cerebral coma is 72 hours from the time of coma onset (Jain et al., 2000[49]). Malaria is an infectious disease transmitted by mosquitos and caused by *Plasmodium* protozoan parasites (Abebaw et al., 2022[2]). These parasites can be transferred through blood transfusions, particularly red blood cell concentrates collected from asymptomatic and parasitemic donors (Mushtaque and Shahjahan, 2015[74]). There are about 100 species in the genus *Anopheles*, but only 50-60 can transmit malaria. In rare cases, infected blood may cause an infection in a person or a fetus (Solomon et al., 2014[103]). Two hosts, a female *Anopheles* mosquito and a human, are essential for the parasite. When a female mosquito bites its prey, the malarial life cycle begins, with the mosquito extracting blood and infecting her with saliva-containing sporozoites (Foley and Tilley, 1998[37]). Malaria parasite invades red blood cells (RBC) where hemoglobin is depleted within and acidic digestive vacuole is released due to heme digestion that is toxic to parasites. The natural parasite protection mechanism consists of ferriprotoporphyrin oxidation, which forms less harmful hemozoin (Navarro et al., 2011[78]). The malaria parasite has a complicated life cycle within the mosquito and human beings (Nureye and Assefa, 2020[81]). Prophylactic antimalarials are used almost exclusively by travellers visiting malaria-endemic countries from developed countries (Saifi et al., 2013[95]). Drug development programs for malaria have evolved over the past decades with pharmaceutical development partnerships (Ashley and Phyo, 2018[10]; Yeung et al., 2010[119]). The current malaria condition and complexities require multiple approaches to overcome parasite drug resistance by employing newer antimalarials (Bhuvaneshwari and Kondaveti, 2015[14]; Monroe et al., 2022[70]). The specific problems of global malaria currently are reflected in the World Malaria Report 2022 (WHO, 2019[115], 2022[116]). This year's World Malaria Day, April 25, has the theme "Harness innovation to reduce malaria disease burden and save lives." After several years of stalling progress, WHO is urging increased investment and innovation in vector control, diagnostics, and drugs to assist nations in eliminating malaria (Lancet, 2022[53]). Antimalarial resistance against parasites leads to the design and development of newer antimalarials (Egwu et al., 2022[35]).

## Life Cycle of Malaria Parasite

*Plasmodium* parasites alternate between sexual and asexual phases in female *Anopheles* mosquitoes and two sequential stages of asexual growth inside two very different types of host cells in vertebrate hosts (Chora et al., 2022[20]). The complex life cycle of the human malaria parasite needs both a human host and an insect host (Gupta, 2015[42]; Doerig et al., 2009[31]). It attenuates its sexual process in mosquitoes between mosquitoes and hosts (Patzewitz et al., 2013[84]). 

In *Anopheles* mosquitoes, *Plasmodium* reproduces sexually, whereas in humans, it reproduces asexually (by cell division in the host cells), first in the liver cells, then in the red blood cells (RBCs) (Neal and Schall, 2014[79]). Fewer parasites are also needed for human-to-mosquito transmission; only 10^3^ of the total 10^8^-10^9^ mature, sexual stages (falciform gametocytes) present in the blood of critically unwell patients must be transferred to the feeding female Anopheles mosquito taking a ~2 μl blood meal (Birkholtz et al., 2022[16]).

**Figure 1:**
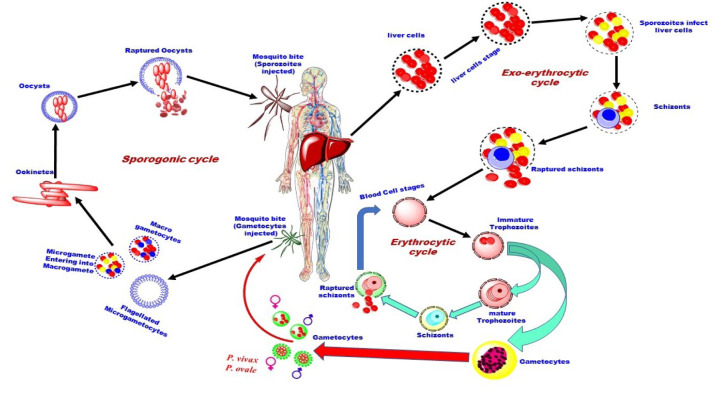
The life cycle of the malaria parasite

A few dozen to several hundred salivary gland-resident, mammalian-infective sporozoites are injected into the skin of the vertebrate host by mosquitoes during a subsequent blood meal. Sporozoites have been infiltrated by gliding motility to enter a blood vessel and then travel through the blood until they arrive at the liver sinusoids. At this site, sporozoites cross the endothelium to enter the liver and then go through numerous hepatocytes and Kupffer cells before productively entering one and forming a parasitophorous vacuole (Chora et al., 2022[20]). The sporozoite-like thread then invades the liver cells. Each sporozoite grows into a schizont within the next 5-15 days, a structure that contains 10,000-30,000 merozoite (Fujioka and Aikawa, 2002[38]). Mature schizont is 30-70 µm large, has no pigment and occupies the entire cell cytoplasm. Hundreds of sporozoites are released into the body cavity or hemocoel over 10-14 days, gradually migrating to the salivary glands where they live and are ready for release at the next blood feeding level (Angrisano et al., 2012[8]). Alternatively, some sporozoites from *P. vivax* and *P. ovale* transform into hypnozoites. Hypnozoites are the form in which the liver remains dormant for months or years. The hypnozoites grow to form schizonts (Figure 1(Fig. 1)) that can cause relapses in infected individuals if they become involved again (Deshpande and Kuppast, 2016[29]). 

Upon rupture of schizonts, Merozoites released from the liver rapidly invade RBCs, where they expand by consuming hemoglobin. Most merozoites undergo the second round of asexual reproduction inside the RBC by forming schizonts, which are once more filled with additional merozoites. The ruptures of the schizont cells and merozoites break out upon maturation. The newly released merozoites invade different RBCs, and their life cycle continues. It completes its life cycle through the mosquito, where some of the merozoites that enter RBCs develop into male and female forms known as gametocytes rather than schizonts through asexual evolution (Solomon et al., 2014[103]). The malarial parasites construct flagella when the male gamete develops in the female mosquito vector's midgut. The ingestion of mosquito-infected malaria blood contributes to the production of microgametes from the terminally arrested gametocytes (Sinden et al., 2010[100]).

### Gametocyte development

The sexual stage of the malaria parasites is called gametocytes. Gametocytes develop in the human host and are transmitted to the vector mosquito to continue its life cycle. Well-coordinated gene activation and silencing sequences facilitate gametocyte formation and activation in the mosquito midgut, both of which are necessary for the parasite to be ready for transmission from the human to the insect host (Hanhsen et al., 2022[44]). The gametocyte is the gamete precursor in the mosquito midgut to the gamete sporogamy. A single mosquito bite can potentially transmit *Plasmodium falciparum* sporozoites, the parasite responsible for human malaria. These sporozoites can initiate exoerythrocytic asexual schizogony in humans (Birkholtz et al., 2022[16]). It is complete in five stages. The five stages of gametocyte development are shown in Table 1(Tab. 1).

The gametocytes reside in the parasitophorous vacuolar membrane within the red blood cell (Bantuchai et al., 2022[12]). Gametocytes are similar to the young asexual trophozoites, and the differentiation of gametocytes takes place in stage II as subpellicular cytoskeleton forms, which are supported by a few microtubules. After stage II development, D-shape gametocytes include similar male and female forms in stage III. Cytoskeleton formation completes by including more symmetrical gametocytes in stage IV and stage V, whereas the host cell appears as a thin, flattened layer around the parasite. The growth and development of *P. falciparum *gametocytes is completed between 8-17 days from merozoite to a mature gametocyte (Singh and Kumar, 2015[101]). The parasites are modified to target red blood cells and quickly multiply, producing waves of parasites that spread throughout the bloodstream. *Plasmodium* parasites consume the host's hemoglobin within red blood cells to gain the amino acid needed to make their proteins (Robert et al., 2002[93]). 

When malaria transitioned from regulation to eradication, stakeholders set treatment and chemoprevention goals despite the limited number of novel compounds entering preclinical research in the preceding decade (Phillips et al., 2017[86]).

## Chemoprophylaxis

Chemotherapy remains one of the most essential tools for treating and controlling malaria (Chilengi et al., 2011[19]). Because of the scope of the parasite's life cycle, its genome codes almost 5300 proteins, several molecules or their fragments extracted for various stages of parasite replication (Patarroyo et al., 2008[83]). Malaria chemotherapy ultimately includes the killing of asexual parasites and supplying the host with supportive treatment to improve the immune system. Quinine, pamaquine, chloroquine and mepacrine were developed prior to World War 2. These were followed by proguanil and amodiaquine (1940s), sulfadoxine (1960s), primaquine and pyrimethamine (1950s), and Artemisinin (1970s). It's essential to keep in mind that there are many viable and cost-effective drugs for the prevention and treatment of malaria and that vaccines can be produced at a reasonable cost if one is formed.

A variety of drugs, such as Mefloquine, Halofantrine, Aablaquine, Pyronaridine, Piperaquine, and derivatives of Artemisinin (including Artemether, Artesunate, and Dihydroartemisinin), were introduced in the 1980s as part of combination therapies. Drug chemoprophylaxis in malaria-endemic areas has proven to be an important preventive technique (Trouiller et al., 2002[109]); despite not typically preventing the later relapses that can happen with *P. vivax* and *P. ovale*, this treatment is effective for both *P. falciparum* and *non*-*falciparum* malaria. At different stages of the *Plasmodium *biological cycle, drugs can work on the pre-erythrocytic liver forms (causal prophylaxis) and the erythrocytic blood forms (suppressive prophylaxis) (Paredes et al., 2006[82]). Antimalarial drugs are vital for treating and chemoprevention of clinical malaria (Amina et al., 2010[7]).

## New Drugs Synthesized for Antimalarial Treatment under Phase Trials

Malaria parasite Artemisinins acquire resistance to Artemisinins and other partner antimalarial drugs. Hence, newer drugs are needed to combat the threat of resistance to antimalarials (Bhuvaneshwari and Kondaveti, 2015[14]). In western Cambodia, 800 km away on the northwestern border of Thailand, rates of treatment failure increase against artesunate and mefloquine. In western Cambodia, *P. falciparum* malaria, which is resistant to Artemisinin, has emerged (Phyo et al., 2012[87]). By preventing parasite transmission between the human host and mosquito vector, the burden of malaria can be demonstrably reduced (Birkholtz et al., 2022[16]). The international effort to control and eradicate malaria is in danger due to *Plasmodium falciparum* developing an increased resistance to Artemisinin derivatives (ART). Although several antimalarial drugs are also available in the market, there is a need to create newer drugs (Gabbiani et al., 2009[39]). The risk of resistance is increasing daily, as has happened previously with chloroquine and sulphadoxine/pyrimethamine-resistant parasites (Ariey et al., 2014[9]). An estimator was used to estimate parasite clearance, which provides a consistent, accurate and reliable estimate of the lag phase and parasite clearance rate. Parasitic clearance can be detected by this early sign of emerging resistance to artemisinin-Artemisinin derivatives and to other compounds of ring-stage or other ring-stage compounds (Flegg et al., 2011[36]). Artemisinin drug resistance in *Plasmodium falciparum *in combination therapy leads to the formation of new drugs (Amartunga et al., 2016[6]). The mechanism of artemisinin resistance against malaria remains unclear; hence, the resistance of parasites against antimalarial combination therapy appears (Pulcini et al., 2013[91]). In malaria-endemic areas, Artemisinin monotherapy should not be used as it requires an extended administration period and may lead to treatment failure (Noedl et al., 2008[80]). It is crucial to remember that numerous efficient and affordable medicines are available to prevent and treat malaria and that vaccines can produce reasonably affordably if one is discovered (Aleshnick et al., 2022[4]). Table 2(Tab. 2) lists the more recent drugs that are undergoing phase trials. There are currently several novel antimalarial candidates in the clinical development stage. Although field studies now routinely assess novel drugs against both *P. falciparum* and *P. vivax*, early clinical trials often only test them against *P. falciparum*. Studies using volunteers infected with malaria have demonstrated to speed up the evaluation of new antimalarial drugs for *P. falciparum*, lowering the period from first-in-human to selecting an efficacious dose (Collins et al., 2022[23]). Focusing on recent developments of the most promising antimalarial candidates in clinical and preclinical phases follows a presentation of the current antimalarial chemotherapy, from quinine to the most recent commercially available drugs (Tisnerat et al., 2022[108]).

### Tafenoquine

Tafenoquine, an analogue of the 8-aminoquinoline primaquine, was given US FDA approval in 2018 for two indications: the prevention of *Plasmodium vivax* malaria relapse and prophylaxis of malaria for up to 6 months. The drug's extended half-life of 14-17 days in humans allows for weekly administration to prevent malaria with just a single dose. The synonym for Tafenoquine is WR 238605 with IUPAC Name: N [ 2,6 Dimethoxy-4-methyl-5[3(trifluoromethyl)phenoxy] quinolin-8-yl] pentane-1,4-diamine.

**Figure 2:**
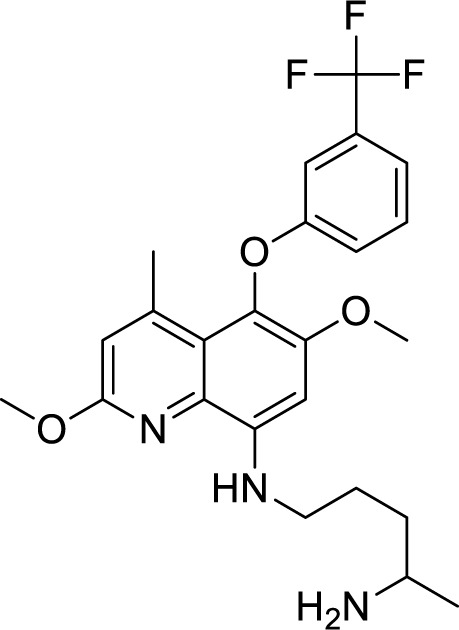
Tafenoquine

Initially formulated as a substitute for primaquine, Tafenoquine (Figure 2(Fig. 2)) was developed to prevent *Plasmodium vivax* relapses. *Plasmodium cynomolgi B* and *Plasmodium fragile* in rhesus monkeys have been tested for blood schizonticidal activity against malaria infections (Marcos et al., 2022[63]). 

In monkeys, treatment with WR 238605 at a dose of 3.16 mg (base)/kg/day for 7 days eliminated infections caused by both parasites. Nine out of twelve monkeys with *Plasmodium cynomolgi* *B* infection and ten out of eleven with *Plasmodium fragile* infection were cured with a lower dose of 1 mg/kg/day (Puri and Dutta, 2003[92]). In addition, the antimalarial drug candidate Ferroquine (FQ, SSR97193) was also evaluated primarily because of its structural similarity. In endemic areas where malaria coexists, this new drug can provide an exciting alternative (Mahajan et al., 2011[62]).

### Phase I trial

The study was conducted in Lambarene, Gabon, a region with a high prevalence of *P. falciparum* malaria between February and July 1999. Tafenoquine, with a therapeutic and safety profile, is a primaquine analogue. It is effective against liver-stage malaria due to its lengthy half-life and parasite activity. Participants were invited from three secondary schools in the area and varied in age from 12 to 20. Glucose-6-phosphate dehydrogenase deficiency, breast feeding, lactation, severe underlying illness, documented HIV infection, QT interval longer than 0.44 s, and a significant departure from the typical variations of serum chemistry or hematological indices (including hemoglobin 8 g/dL and platelet count < 80) were the contraindications.

### Design and procedure

**Figure 3:**
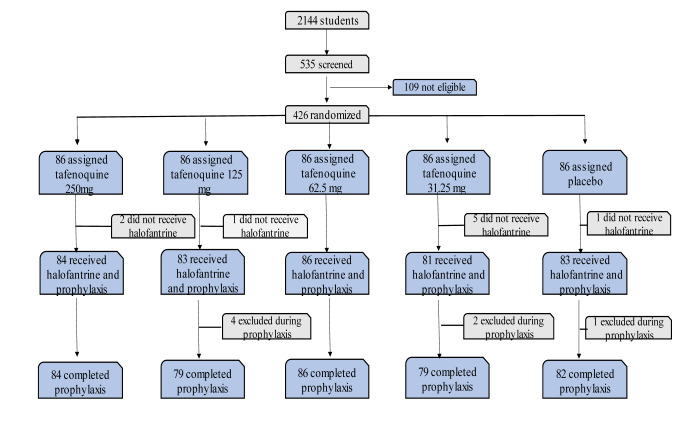
Design of study of the drug in humans

Screening study was performed by 3-day halofantrine radical cure, a 3-day Tafenoquine or placebo prophylaxis, and a 70-day follow-up period. During the screening, all volunteers received a physical examination, an electrocardiogram was recorded. A venous blood sample was collected for measuring glucose-6-phosphate dehydrogenase activity, obtaining a complete blood count, and testing for pregnancy in female subjects 535 students took part in the study, which involved inviting 2144 students to participate out of which 426 qualifying patients were given Tafenoquine at random (250 mg, 125 mg, 62-5 mg, or 31-25 mg) (Figure 3(Fig. 3)) depicted the design and procedure.

In Lambarene, Gabon, *Plasmodium falciparum* malaria is endemic, 2144 participants between 12 and 20 were invited to participate. To 426 eligible participants and a total of 535 individuals, Tafenoquine (250 mg, 125 mg, 62.5 mg, or 31.25 mg) or placebo was randomly assigned each day for three days.

Initial curative care with halofantrine was obtained by 417, and 410 completed the prophylaxis regimen assigned. The drug also showed some adverse events in patients when given for 28 days (Table 3(Tab. 3)). The effects in no. of patients vary with the dose concentration. The common adverse effects shown are headache, abdominal discomfort, fever, diarrhea, disorientation, exanthema and weariness. Two groups of patients-one receiving Tafenoquine and the other a placebo-are formed. In the placebo group, there are 2 patients with headache, 1 with abdominal pain, 1 with fever, and 1 with dizziness. In the Tafenoquine group, the number of patients varies with the doses. In 31.25 gm dose of Tafenoquine, the number of patients with abdominal pain is 3, 2 with dizziness and 1 with exanthema. In a 62.5 gm dose of Tafenoquine, patients with headache is 1, 4 with abdominal pain, 3 with fever, 3 with dizziness, 1 with diarrhea and 1 with fatigue. In 125 gm dose, there are 2 patients with headache, 1 with abdominal pain, 1 with fever and 1 with diarrhea. In the 250 gm dose, 5 patients are with headache, 7 with abdominal pain, 3 with fever and 1 with a 250 gm dose, 5 patients have headaches, 7 with abdominal pain, 3 with fever, and 1 with fatigue. The report showed that total of 7 patients in the placebo group, 6 in 31.25 gm dose group, 26 in 62.5 gm dose group, 9 in 125 gm dose group and 17 in 250 gm dose group had adverse effects. 

With a dosage of 62.5 mg of Tafenoquine per day for 3 days, the drug exhibits long-term activity, and no reinfection happened within the first 7 weeks. At this point, a 3-day course of 250 mg serves as an adequate dosing schedule for chemoprophylaxis in a population (Lell et al., 2000[56]). 

On day 56, the report of cases is presented in Table 4(Tab. 4), comparing results between the placebo group and the Tafenoquine group based on cases of parasitemia, individual age in years, and the percentage of protective cases.

Eight positive blood smears were recorded by day 56 (four/82 placebo participants and four/79 Tafenoquine 31.25 mg group). Table 5(Tab. 5) shows the report of patients on day 77 with cases of parasitemia, incidence rate and percentage of protective efficacy. 34 positive blood smear results were noted by day 77 (14/82 placebo; 16/79 Tafenoquine 31.25 mg; 3/86 Tafenoquine 62.5 mg; 1/79 Tafenoquine 125 mg; none/84 Tafenoquine 250 mg). Tafenoquine is a primaquine substitute with a better therapeutic and safety profile.

Even at its highest dosage, Tafenoquine was well taken. Previous research administered Tafenoquine at doses up to 600 mg, demonstrating its safety and good tolerability.

Out of 2144 students, 535 were screened. 109 were ineligible (60 had glucose-6-phosphate dehydrogenase insufficiency, 14 had positive pregnancy tests, 12 had excessive alanine aminotransferase activity, 4 had anaemia, 8 had withdrawal of consent, 6 had no follow-up, 5 were omitted for other reasons).

The research regimens were allocated to 426 people randomly. Nine people were omitted until initial curative care was obtained as their consent was removed. Of the 417 people who received halofantrine, 107 (26 %) already had parasitaemia, with a low count (geometric mean 258 µL) of *P. falciparum* in most cases, or about 91 %. One week after receiving halofantrine treatment, no participant had parasitemia, as determined by a thick blood smear at the start of the prophylaxis process.

During prophylaxis, seven persons were omitted because of procedure infringement (three cases), follow-up failure (three), or pregnancy (one). In the follow-up stage, the remaining 410 participants entered. The five study groups' demographic, clinical, hematological, and biochemical feature distributions were identical. Positive blood smears were detected from day 35 onwards during the weekly visits. The 3-day, low-dose halofantrine regimen was highly effective since no one in the placebo group developed parasites in the four weeks following radical therapy. From day 56 through the primary endpoint, eight positive smears were identified. The lowest dose of Tafenoquine was given to four of these individuals and a placebo to four individuals. 34 positive blood smears were registered on day 77 of the secondary endpoint. On day 70, a subject who got a dose of more than 31.25 mg of Tafenoquine showed the first positive smear. No one in the population receiving Tafenoquine 250 mg developed malaria at any point during the follow-up period.

The parasite levels were generally low during follow-up (geometric mean 245/ μL), with a *P. falciparum *to *P. malariae* or *P. ovale* (three) ratio typical of the research location. At day 77, all Tafenoquine doses of more than 31.25 mg provided significant safety compared to placebo (Table 3(Tab. 3)). Despite the fact that no infections were detected up to day 56, the protective efficacy was not statistically significant due to the low numbers. 24 participants completed the analysis of the study; 24 were omitted due to pregnancy (ten cases), breach of the procedure (nine), or follow-up failure (five).

In three cases of pregnancy, the test result was later discovered to be a false positive. 180 adverse effects were recorded during the study. In general, they were light and self-restricting, and none were rated as severe. After the drug intake study was finished, adverse case analysis was conducted for one week (29 participants), four weeks (65 participants), and at the end of the study (170 events). However, the incidence of stomach pain was higher in the Tafenoquine group than in the placebo group, with the 250 mg Tafenoquine group having the highest frequency. No other symptoms have been shown to be significantly related to the drug under study. The hemoglobin concentrations in the 250 mg Tafenoquine group on day 28 were slightly (0.4 g/dL), but considerably lower than at screening. Packed-cell volume and biochemical indicators indicated minimal fluctuation at various time intervals, as there was slight variation between groups, and everything was within normal ranges. On days 14 and 28, one member of the placebo group reported symptomatic increases in alanine aminotransferase activity, which naturally reverted to baseline on day 77 (Lell et al., 2000[56]). 

### Phase II trials

The study was performed between Sept 19, 2011 and March 25, 2013. Men and women were enrolled in this double-blind, randomized, dose-ranging phase IIb study. Parasite density mono-infection was found to be > 100 to < 100 000 per μL of blood with *P. vivax*. The enrolled volunteers at seven locations in Brazil, Peru, India, and Thailand were from community health centers and hospitals, and they had to be at least 16 years old. Patients with an activity of less than 70 % of the glucose-6-phosphate dehydrogenase enzyme were omitted. Eligible patients received chloroquine (days 1-3) and were randomly allocated to receive a single dose of 50 mg, 100 mg, 300 mg or 600 mg of Tafenoquine (1:1:1:1:1:1). Before beginning the trials; the baseline parasite count was between 7500 and > 7500 per L of blood. A treatment group (50 mg [n=55], 100 mg [n=57], 300 mg [n=57], 600 mg [n=56], chloroquine plus Tafenoquine) was assigned at random to 329 patients.

**Figure 4:**
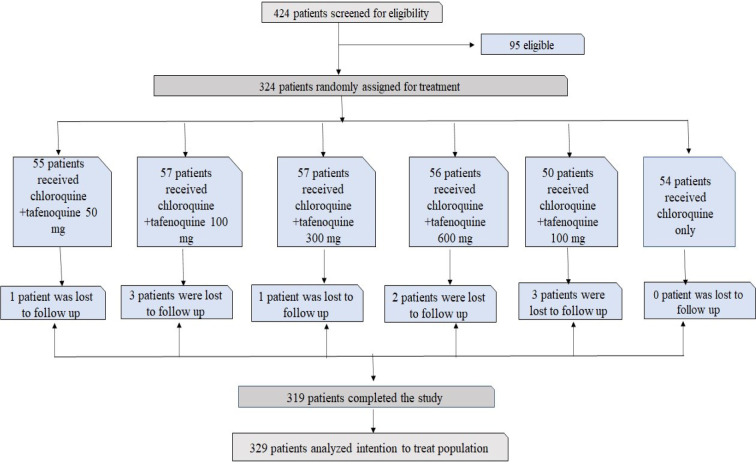
Chart of the patients for further phase trial study

Figure 4(Fig. 4) shows the patients enrolled in the phase trial study. Of the 424 patients screened for eligibility, 95 were ineligible: 21 patients had elevated aspartate aminotransferase, 18 subjects did not meet the G6PD eligibility criteria, 17 subjects had no *P. vivax* parasites, 8 had low *P. vivax* parasites, 5 with concomitant disease, 4 without consent, 3 with unmet need for childbearing, 3 with drug unavailability, 2 with mixed malaria infection, 2 subjects did not meet hemoglobin requirements, 1 with severe malaria, 1 with adverse event prior to randomization. Phase trial studies are shown in Figure 4(Fig. 4). The number of patients varies in each cohort. The drug Tafenoquine is employed in combination with chloroquine where doses vary as 50 mg, 100 mg, 300 mg, 600 mg, chloroquine plus primaquine and chloroquine only. In this study, about 319 patients completed the study, and 329 patients were further analyzed for treatment. The results of the efficacy of Tafenoquine up to 6 months are shown in Table 6(Tab. 6). The % efficacy of the drug increases with an increase in dose concentration.

Both Tafenoquine 300 mg and 600 mg, as well as primaquine (treatment difference of 39 % to 9 % [21-59], p=0.0004), were more effective than chloroquine alone (treatment differences of 51 % to 7 % [95 % CI 35-69], p<0.0001, Tafenoquine 300 mg, and 54 % to 55 % [38-71], p<0.0001, Tafenoquine 600 mg, respectively). Male and female patients who presented with malaria symptoms of age 16 or more are eligible for further studies. Inclusion criteria included uncomplicated *P. vivax* mono-infection confirmed by microscopy and an asexual parasite density of more than 100 μL blood but less than 100,000 μL.

A combination of the drug with Tafenoquine at different doses improved the efficacy of the drug. The doses of the drug are shown in Table 7(Tab. 7). Using the Kalpan-Meier approach, the relapse-free efficacy at six months of therapy was determined. The number of patients for each cohort was 55 for the 50 mg dose, 57 for the 100m g dose, 57 for the 300 mg, and 56 for the 600 mg dose. 

ND stands for "not determined," as the more excellent dose comparison was insignificant. The efficacy of Tafenoquine and chloroquine in combination was increased with the increase in dose, at 50 mg it is 57.7 %; at 100 mg it is 54.1 %; at 300 mg it is 89.2 %; at 600 mg it is 91.9 %. The median parasite time for clearance of fever and gametocytes were shown in (Table 8(Tab. 8)). The clearance time was calculated as parasite clearance time in hours, fever clearance time in hours, and gametocyte clearance time in days. The parasite clearance time of the drug was 45, 43, 42, 47 hours for 50 mg, 100 mg, 300 mg, and 600 mg doses, respectively (Llanos-Cuentas et al., 2014[60]).

Fever clearance time was 11, 6.5, 15, 19 hours for 50 mg, 100 mg, 300 mg, and 600 mg, respectively. Gametocyte clearance time varies by dose and is measured in days. The incidence of diarrhea was higher in the Tafenoquine 600 mg group (nine [16 %] of 56) than in the other groups.

According to Kaplan-Meier estimates, the Tafenoquine relapse-free efficacy after therapy for 6 months was shown in Table 9(Tab. 9). The % efficacy in Peru was 22 %, 24 %, and 27 %, according to 50 mg, 100 mg, 300 mg, and 600 mg doses, respectively. In Thailand, the % efficacy was 16 %, 16 %, 19 %, 16 %. In Brazil it was 6 %, 6 %, 6 %, 7 % and in India it was 11 %, 11 %, 9 %, 10 %. After chloroquine treatment, a single 600-mg dose of Tafenoquine will effectively avoid a *P.vivax* malaria relapse (Schrader et al., 2012[98]). Tafenoquine (WR238605) is an 8-aminoquinoline discovered at the Walter Reed Army Research Institute, USA, in 1978 (Pinedo-Cancino et al., 2022[90]).

Tafenoquine's safety and effectiveness were examined in a phase II, double-blind, placebo-controlled, randomized study of individuals with mild-to-moderate COVID-19 disease. 86 patients with mild-moderate COVID-19 disease participated in this randomized, double-blind, placebo-controlled trial, run at 15 outpatient locations across the United States between March 1 and September 10, 2021. Over 28 days, mild-moderate COVID-19 patients received Tafenoquine at doses of 200 mg once daily on days 1, 2, 3, and 10. Both safety and effectiveness of this treatment were assessed. The main outcome was clinical COVID-19 symptom recovery on Day 14 as measured by a mild or absent cough, a respiratory rate of less than 24 beats per minute, and no fever or shortness of breath. The trial was discontinued early and unblinded after a fruitful futility analysis after n = 86 patients out of a target; n = 275 were randomized to aid in planning studies. The percentage of patients who had not recovered by Day 14 had numerically dropped by 47 % in the PP population [5/42 vs. 9/41, P = 0.25] and by 27 % in the ITT population [8/45 vs. 10/42 not recovered in the Tafenoquine and placebo arms, P = 0.60]. All Tafenoquine patients recovered among those who reported responses in an electronic diary on Day 28, but up to 12 % of placebo patients showed persistent dyspnea. In the Tafenoquine group, the time to complete clinical recovery from COVID-19 symptoms was accelerated by roughly 2-2.5 days. The placebo group had two hospitalizations due to COVID-19, while the Tafenoquine group had one. In the Tafenoquine arm, 8.4 % of people experienced mild drug-related side effects [vs. 2.4 per cent in the placebo]. In this trial, Tafenoquine therapy in outpatients with mild to moderate COVID-19 disease may provide therapeutic benefits, even if this trial was underpowered for the primary goal due to its early conclusion. In VERO and human epithelial cells, Tafenoquine had an EC50/90s against SARS-CoV-2 of 2.6/5.1 M and 8.6/17 M, respectively, and was at least a thousand times more potent than other aminoquinoline antimalarials when protein binding was taken into account (Dow and Smith, 2022[32]).

### Phase III trials

**Figure 5:**
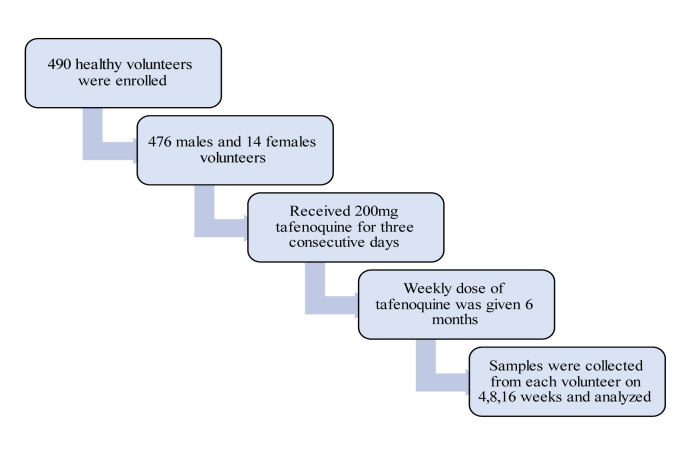
Phase trial studies and sampling

The phase III trials' study assesses safety, tolerability and efficacy of Tafenoquine in adults, the only approved clinical treatment for *Plasmodium. *After oral administration, Tafenoquine is steadily absorbed, reaching its peak plasma concentrations in fasted subjects around 12 hours after the dose. The half-life of Tafenoquine in the body is around two weeks. It has a large volume of tissue distribution and low clearance and is broadly dispersed throughout tissues. The soldiers were on a 200 mg weekly prescription of Tafenoquine, and blood samples were obtained for drug analysis on four occasions (Llanos-Cuentas et al., 2022[61]). Tafenoquine safety, acceptability, and effectiveness for weekly malaria prophylaxis in Australian soldiers were the primary goals of the clinical research, which was designed as a prospective, randomized, double-blind comparative study. The participants were sent to East Timor for 6 months on peacekeeping duties. Typical hematological and biochemical values, a thorough physical examination, and a complete medical history were all considered positive. They had to be healthy, willing, and able to follow the study's protocol while providing written informed consent (Mayoka et al., 2022[64]). The study is shown in Figure 5(Fig. 5).

The individuals were given an oral weekly dose of 200 mg Tafenoquine for 6 months after receiving a loading dose of 200 mg Tafenoquine daily for three consecutive days. An opaque Swedish Orange size 1 firm gelatin capsule (Capsugel) with 200 mg of Tafenoquine (pure free base) served as the dosing form (Charles et al., 2007[17]).

The post-dosing of Tafenoquine for six months was primarily metabolized by CYP2D6 (Jean et al., 2016[51]). The samples were collected on predetermined days of weeks 4, 8, and 16. 

Days 1 (early post-dose; absorption phase), 3, and 5 (72-120 h post-dose), as well as days 7 (predose; trough phase), were predetermined. All mild adverse events occurred 1 to 24 days after the start of Tafenoquine treatment, while diarrhea and abdominal pain were only noted in a single subject with serious effects 2 days after the start of Tafenoquine treatment (Charles et al., 2007[17]).

In non-immune Caucasian subjects, after 6 months of prophylaxis, Tafenoquine at 200 mg once a week is safe and well tolerated (Nasveld et al., 2010[76]). Tafenoquine's low dose also showed the same effect as 600 mg of its doses in the treatment of *Plasmodium vivax* malaria (Walsh et al., 2004[111]). Tafenoquine, a primaquine-related long-acting 8-aminoquinoline drug, was approved by the Food and Drug Administration (FDA) for anti-relapse therapy (Krintafel) on July 20, 2018, and chemoprophylaxis (Arakoda) on August 8, 2018. This research explores the evidence for the effectiveness and protection of Tafenoquine and offers recommendations from the CDC for clinicians prescribing chemoprophylaxis for endemic malaria travellers and treating malaria (Haston et al., 2019[45]).

The trial's last follow-up appointment was on December 23, 2021; ClinicalTrials.gov has the trial's registration number, NCT04609098. Tafenoquine has received approval as a preventive and radical treatment for *Plasmodium vivax* infection, although it is unknown how well it will prevent *Plasmodium falciparum* transmission.

Participants in a single-blind, phase 2 randomized controlled trial were enlisted at the University of Bamako in Mali's Clinical Research Unit of the Malaria Research and Training Center. Participants needed to be between ages of 12 and 50, have asymptomatic *P. falciparum* gametocyte carriage detected by microscopy, weigh 80 kg or less, and not have any clinical symptoms of malaria, such as fever. The dihydro-Artemisinin-piperaquine conventional treatment, or dihydro-Artemisinin-piperaquine plus a single dose of Tafenoquine (in solution) at a final dosage of 0.42 mg/kg, 0.83 mg/kg, or 1.66 mg/kg were given to participants in a 1:1:1:1 random order. A computer-generated randomization list was used for the randomization, which was then covered up with sealed, opaque envelopes. According to the manufacturer's recommendations, dihydro-Artemisinin-piperaquine was given orally over three days (days 0, 1, and 2). On day 0, in addition to the initial dose of dihydro-Artemisinin-piperaquine, a single dose of Tafenoquine was also given as an oral solution. The primary endpoint was the median percentage change in mosquito infection rate 7 days after treatment compared to baseline, which was evaluated in the population that adhered to the procedure.

Adverse event incidence and frequency were safety endpoints. A total of 1,091 people were screened for eligibility from October 29 to November 25, 2020, and 80 were enrolled and randomly allocated (20 per treatment group). Before treatment, 12 to 50 % of mosquitoes were infected by 53 (66 %) of the participants, a median rate (IQR: 3.64-35.00). Following administration of dihydro-Artemisinin-piperaquine alone, the within-group reduction in mosquito infection rate was 79-95 % (IQR 57.15-100; p=0.0005 for difference from baseline), 100 % (98.36-100; p=0.0005) following administration of dihydroArtemisinin-piperaquine plus Tafenoquine 0.42 mg/kg, 100 % (100-100; p=0.0001). Throughout the trial, 94 adverse events were reported by 55 (or 69 %) of the 80 participants; 86 (or 92 %) of these occurrences were deemed mild, seven (7 %) as moderate, and one (1 %) as severe. Mild to moderate headaches were the most typical treatment-related adverse event, occurring in 15 (19 %) participants (dihydro-Artemisinin-piperaquine, n=2, dihydro-Artemisinin-piperaquine with Tafenoquine, 0.42 mg/kg n=6, 0.83 mg/kg n=3, and 1.66 mg/kg n=4). There were no significant adverse outcomes. There were no discernible differences between treatment groups in the frequency of any adverse events (p=0.73) or adverse events associated with the treatment (p=0.62). Tafenoquine improved *P. falciparum* gametocyte clearance and was well tolerated at all dosages. Compared to dihydro-Artemisinin-piperaquine alone, all Tafenoquine dosages exhibited superior transmission reduction at day 7 (Stone et al., 2022[105]).

Tafenoquine trial registration was completed in the Australian New Zealand Clinical Trials Registry using funding from the QIMR Berghofer Medical Research Institute (ACTRN12620000995976). Tafenoquine shows effectiveness against all malaria parasite stages and could be helpful as a substance that prevents transmission. Erythrocytes infected with *P. falciparum* 3D7 were given to healthy persons as an injection on day 0. In order to eliminate asexual parasitemia and promote gametocyte growth, piperaquine was given on days 9 and 11. On day 25, a single 50 mg oral dosage of Tafenoquine was given. Enriched membrane feeding experiments were used to determine transmission before the dose and at 1, 4, and 7 days after the treatment. The administration of artemether-lumefantrine followed the final assay followed the final assay. Findings included a decline in post-Tafenoquine gametocytemia, mosquito infection, and safety standards. Six subjects were enrolled, and all were pre-Tafenoquine infective to mosquitoes, with a median oocyst positivity of 86 % (range: 22-98) and sporozoite positivity of 57 % (range: 4-92). The median oocyst and sporozoite positive rate decreased by 35 % (IQR: 16-46) and 52 % (IQR: 40-62) on day 4 and 81 % (IQR: 36-92) and 77 % (IQR: 52-98) on day 7 post-Tafenoquine, respectively. Post-Tafenoquine, there was no detectable decrease in gametocyte density. No important participant safety issues were found (Webster et al., 2023[112]), as Tafenoquine may lower the prevalence of *P. vivax* malaria (Suh et al., 2022[106]).

The studies for Tafenoquine were continued in 2021, supporting the safety of extended 52-week Tafenoquine prophylaxis. The analyses were performed under clinical trial registration Number/ClinicalTrials.gov Identifier NCT03320174. 600 healthy adults participated in this randomized, double-blind, placebo-controlled experiment. For 52 weeks, eligible participants were randomized 1:1 to receive either Tafenoquine 200 mg weekly (antimalarial prophylactic regimen) or placebo. Safety inspections were scheduled for Weeks 4, 12, 24, 52 (dosing completed), and 64. Ophthalmic alterations, general and neuropsychiatric adverse events (AEs), and laboratory value changes were all monitored for safety.

Tafenoquine, like other cationic amphiphilic drugs (for example, chloroquine), can cause an intracellular accumulation of phospholipids in the cornea, resulting in whorl-like patterns ("vortex keratopathy" or "cornea verticillata") that disappear after treatment is discontinued. Similar phospholipid accumulations in the retina could theoretically produce retinopathy and other visual abnormalities, but no such consequences have been found. Although the US FDA considers Tafenoquine to be contraindicated in people with psychotic disorders or current psychotic symptoms, the US National Academy of Sciences [NAS] concluded that existing data provide insufficient/inadequate evidence for a link between Tafenoquine prophylaxis and psychiatric outcomes. The NAS suggested that further study was warranted, noting possible confounding and other potential biases in previous studies and the methodology for collecting psychiatric AEs (National Academies of Sciences, Engineering, and Medicine, 2020[77]).

Five significant clinical trials provided pre-approval safety information for the preventive weekly dose regimen of Tafenoquine (Arakoda™, Kodatef®) in the US and Australia. A randomized, double-blind, active-controlled trial comparing Tafenoquine to mefloquine in non-immune Australian soldiers deployed to Timor Leste was conducted, along with three separate randomized, double-masked, placebo-controlled trials of Tafenoquine in residents of malaria-endemic regions. Additionally, a 6-month randomized, double-blind, placebo-controlled safety study of Tafenoquine was carried out in healthy adults in either the United States or the United Kingdom. Pregnant women and G6PD-deficient patients were excluded from these trials. Furthermore, three investigations excluded participants with a history of psychiatric illnesses. For ethical and operational considerations, the trial with non-immune deployed soldiers did not include a placebo control group. None of these trials dosed Tafenoquine beyond six months, resulting in a six-month dosing period. The current study examined the safety of the approved weekly Tafenoquine prophylactic regimen for beyond 6 months - in this case, 52 weeks/1 year. This was a randomized, double-blind, placebo-controlled trial in 600 healthy adults (age 18-55 years) male and non-pregnant female volunteers living in Australia or the US and age-matched to the Australian Defence Force (ADF) study subjects. The study took place at 3 study sites: Linear Clinical Research and the Lions Eye Institute, Nedlands, WA, 6009, Australia; Retina Consultants of Southern Colorado, Colorado Springs, CO, 80909, USA; and Valley Retina Institute, McAllen, TX, 78503, USA. Subjects were recruited from the study site's approved database of healthy subjects or in response to ERB-approved advertisements. This study fulfils a specific post-marketing directive provided by the US FDA, submitted under the risk management plan (RMP) approved by the Australian Therapeutic Goods Administration (TGA). Compared to the Placebo Group (19 %, p = 0.308), the Tafenoquine Group (18.2 %) had fewer participants with a Serious Ophthalmic Safety Event as defined by protocol. The percentages of participants in the Tafenoquine Group (91.0 %) versus the Placebo Group (89.9 %, p = 0.65) who experienced at least one adverse event did not vary significantly. Reversible cornea verticillata (54.5 %) and nausea (13.1 %) were frequent adverse events (AEs) observed at a considerably higher incidence for Tafenoquine, resulting in 0.0 % and 0.7 % discontinuations, respectively. In both research groups, psychiatric adverse events (AEs) happened at comparable rates. Hemoglobin, methemoglobin, creatinine, and blood urea nitrogen (BUN) were found to have reversible alterations.

### KAE609

**Figure 6:**
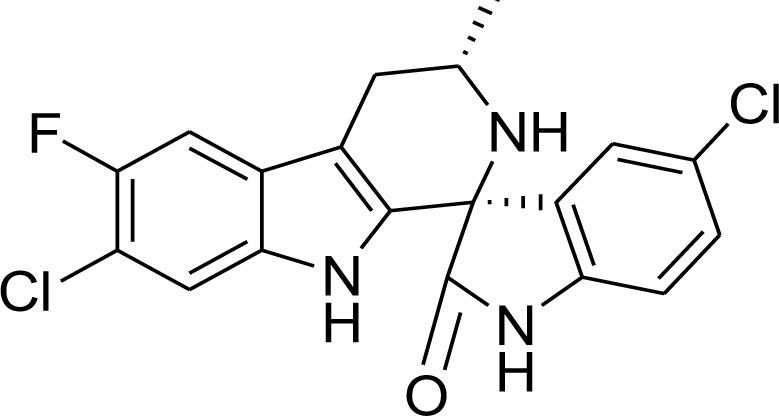
Cipargamin

Cipargamin (KAE609), a novel antimalarial drug now in advanced stages of research, exhibits encouraging outcomes (Dennis et al., 2018[28]). It reduces *anopheline* mosquito oocyst formation and *P. falciparum* gametocytogenesis. They also exhibit robust asexual blood stage behaviors. Cipargamin, NITD609, and GNF609 are all synonyms (Yipsirimetee et al., 2022[120]). The existence of this mutation will dramatically impact the maximum general use of Cipargamin (Figure 6(Fig. 6)) in terms of policy. The Novartis library of 12,000 natural and synthetic compounds was searched for *P. falciparum*-active molecules before NITD609 was discovered. 275 compounds were reported for the first screening, and this list was categorized into 17 possible compounds (Huskey et al., 2016[47]).

Cipargamin, a Spiroindolone derivative, is used to treat malaria. A synthetic antimalarial drug in the spiroindolone class is Cipargamin. The *P. falciparum* P-type ATPase 4 (PfATP4) Na+ efflux transporter on the plasma membrane of malaria parasites serves as the drug's primary target and the primary mechanism of action (Yipsirimetee et al., 2022[120]). KAE609[(1'R,3'S)­5,7'­dichloro­6'­ fluoro­3'­methyl­2',3',4',9'­tetrahydrospiro- [indoline­3,1'­pyridol [3,4­b]indol]­2­one] is a potent schizonticidal agent with quick action that is being tested in clinical trials for the treatment of malaria. The KAE609 is primarily metabolized by biliary secretions (Huskey et al., 2016[47]). From a phenotypic screen on wild-type *Plasmodium falciparum*, *in vitro* experiments revealed the antiplasmodial activity of a series of racemic spiroazepineindoles (Rottmann et al., 2010[94]). KAE609 demonstrates rapid clearance of malaria-infected RBC cells. In infected red blood cells (RBCs), exposure to KAE609 induces osmotic dysregulation, leading to a transformation into inflexible and spherical shapes. This effect is achieved by disrupting the sodium efflux pump of the malaria parasite. In phase II clinical trials, the drug demonstrates high activity in individuals with the disease. KAE609 exhibits rapid clearance, typically taking less than 1 hour, as opposed to the geometric mean clearance time of 2.6 hours observed with artesunate treatment for uncomplicated falciparum malaria. KAE609 shows high activity on rings and increased activity on ring-stage parasites of *Plasmodium vivax* and *P. falciparum* (Zhang et al., 2016[121]). Cipargamin is a potent ATPase (adenosine triphosphate) inhibitor. When *P. falciparum* enters the uninfected erythrocytes, it passes from the Na^+^ K^+^ pump of blood plasma and turns from high [Na^+^] / low[K^+^] to low [Na^+^] / high[K^+^]. NITD 609 acts by disrupting the Na^+ ^regulation pump in the parasites. The high-fat food intake did not affect how much KAE609 was absorbed. However, it did cause the C_max _to drop by about 27 %. KAE609 was barely eliminated via urine in unaltered form, typically less than or equivalent to 0.01 % of the dose taken. The drug was excreted in urine over a 48-hour post-dose span (Rottmann et al., 2010[94]). The first new antimalarial chemotype in two decades is KAE609, a spiro tetrahydro-carboline. High throughput phenotypic screening was used to find this class.

### Phase I 

It was performed in the Q-Pharm Phase I unit (Brisbane, Australia) from 16 November 2010 to 4 October 2011. A single-center, double-blind, randomized, placebo-controlled experiment was used in the investigation. It is divided into two parts: Part 1 includes single-ascending dose (SAD) cohorts with various doses of KAE609 (1 mg, 3 mg, 10 mg, 30 mg, 100 mg, 200 mg, and 300 mg), as well as one food impact cohort (30 mg) with 4 to 10 participants. In Table 10(Tab. 10), the evaluation study was displayed. It had a maximum screening time of 28 days.

***Part 2***- Multiple ascending dose (MAD) cohorts with doses of 10 mg, 30 mg, 60 mg, 100 mg, and 150 mg once daily for three days are used in part 2 investigations, and each cohort consists of 8 participants. Additionally, a maximum 28-day screening period was included. The study was conducted as a baseline, and treatment and the complete evaluation were conducted between days 9 and 11.

The drug was orally administered with 180-350 ml of water. 94 healthy male and female subjects are involved in the study: 46 subjects in Part 1 and 48 subjects in Part 2 (Leong et al., 2014[58]). The Swiss Tropical Institute, the Biomedical Primate Center, the Novartis Research Foundation (GNF), and the Novartis Institute for Tropical Diseases in Singapore collaborated to develop the molecule. Cipargamin wins the MMV Project of the Year (McCarthy et al., 2021[65]). In June 2012, 21 patients received a proof-of-concept injection of one of the significant malaria-causing parasites. A clinical study was conducted near Thailand's Burma Border in Bangkok and Mae Sot, where resistance to current therapies has been documented. In the New England Journal of Medicine, Novartis released research on KAE609 with simple *Plasmodium vivax* and *P. falciparum* on July 30, 2014. The study demonstrated that the parasites were easily removed within 12 hours. 

Oral administration of the resulting synthesized spirotetrahydro-β-carboline candidate KAE609 resulted in a single dose cure in a *Plasmodium berghei* blood stage animal model. Men and women aged 20 to 60 years with a body weight of 40 to 90 kg and a history of fever or febrile illnesses were eligible patients. From January 10 to June 22, 2012, 21 participants participated in the open-label study. 10 individuals had *P. vivax* malaria, and 11 had *P. falciparum* malaria among the 21 patients. Baseline densities ranged from 6816 to 53,082 per cubic millimeter for *P. vivax* and 4139 to 63745 per cubic millimeter for *P. falciparum*. As part of an open-label phase 2 study, three centers in Thailand examined the antimalarial efficaciousness, safety, and adverse event profile of KAE609 at a dose of 30 mg/day for three days. The median parasite clearance time for *P. vivax *patients was 8-16 hrs, and for *P. falciparum *it was 10-16 hrs. The mean terminal half-life for eliminating KAE609 was 20.8 hrs (White et al., 2014[114]). It also appeared to be safe and well tolerated and showed some tantalizing effects, which suggest it could not only kill blood-stage parasites but could have blocked the transmission of parasites (McCarthy et al., 2021[65]). In clinical practice, combination therapy increases the useful therapeutic impact of individual drugs and medicines. Clinical trials have shown outstanding therapeutic efficacy (McCarthy et al., 2016[66][67]). Parasites were administered with the Spiroindolone compound KAE609, NITD609 or Cipargamin in the fluctuation assay. In response to KAE609, the polymorphic nature of Pfatp4 has helped us to measure mutation rates unique to KAE609 (Lee and Fidock, 2016[55]).

### Pharmacokinetics of KAE609

The plasma concentrations were maximal in the median of 3 hours (C_max_). The half-life of KAE609 after the terminal dose was 20.8 hrs. The pharmacokinetic parameters, such as mean, coefficient of variation, AUC_max_, T_max_, accumulation ratio, and terminal half-life elimination, were calculated for Day 2 and Day 3. The pharmacokinetic parameters of KAE609 on Days 1 and 3 are shown in Table 11(Tab. 11).

KAE609 provides breakthrough treatment for the elimination of malaria. It shows a fast onset of action and potentially reduced parasitemia. It was the only tested drug with single-dose cure efficacy (Diagana, 2015[30]). KAE609 was well absorbed, and such low parent compound levels (about 11 % of the dose) were contained in the faeces extensively metabolized. The liver primarily handled it (about 93 % of the dose). The primary metabolite in the plasma of rat and dog faeces was M18, an oxidative metabolite. KAE609 has been metabolized in the liver, forming a prominent M23 metabolite in human hepatocytes.

### Phase II

A phase II trial is underway in 2021 for effectiveness against antimalaria. Guidelines for Good Clinical Practice (ICH E6) were followed in the trial per the International Conference on Harmonization. The clinical trial (NCT03334747) is listed on ClinicalTrials.gov. In sub-Saharan Africa, adult patients with uncomplicated *Plasmodium falciparum *malaria participated in a phase II, multicenter, randomized, open-label, dose-escalation study. Artemether-lumefantrine was used as a control during the three days of Cipargamin monotherapy, which included single doses of up to 150 mg or up to 50 mg once a day. PCT (parasite clearance time) and ACPR (adequate clinical and parasitological response) at 14 and 28 days (both PCR-corrected and uncorrected) were important effectiveness objectives. Pharmacokinetics and molecular markers of drug resistance were also assessed.

Individuals who met the eligibility requirements were those who were at least 18 years old, weighed at least 45 kg, and had microscopic evidence of acute, uncomplicated *P. falciparum* malaria (parasitemia 500-50.000/μL, axillary temperature ≥37.5 ºC, oral/tympanic/rectal temperature ≥38.0 ºC, or fever history within the previous 24 hours). World Health Organization (WHO) criteria for severe malaria and mixed *Plasmodium *infections were excluded. With a median parasite clearance time of 8 hours compared to 24 hours for artemether-lumefantrine, all single or repeated Cipargamin dosages ≥50 mg were linked to rapid parasite clearance. For every dosage of Cipargamin, the PCR-corrected ACPR was >75 % and 65 %, respectively, at 14 and 28 days. G358S, a therapy-emerging mutation in the Pfatp4 gene, was detected in 65 % of treatment failures. Pharmacokinetic parameters were consistent with previous data, and the dose was approximately dose-proportional.

**Figure 7:**
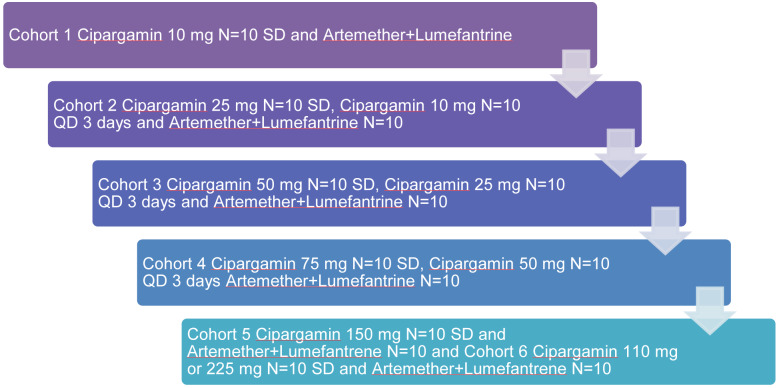
Cohorts with maximum Cipargamin dosages

Five cohorts of patients received treatment with Cipargamin at escalating single or multiple doses (Figure 7(Fig. 7)). The initial design included the enrollment of four cohorts, with the maximal Cipargamin dosages being 75 mg as a single dose and 50 mg once daily for three days. An optional sixth cohort, in which patients would receive 110 or 225 mg single doses of Cipargamin, contingent on hepatic safety in cohort 5, and cohort 5 (a 150 mg single dosage) were introduced in a protocol amendment while the experiment was still in progress. If no added benefit was expected from the 225 mg dose, the trial could be stopped after cohort 5. Cipargamin doses in the first two cohorts were potentially subtherapeutic, so the minimum number (N=10) of patients needed to assess the primary objective was included. Subsequent cohorts were targeted to enroll 20 Cipargamin patients each. It was planned to treat 10 patients per cohort with artemether-lumefantrine (80/480 mg, twice daily for 3 days; Novartis Pharma AG) as an active comparator.

It was found that people with uncomplicated *P. falciparum* malaria showed >65 % PCR-corrected ACPR at 28 days after single doses of Cipargamin and that recrudescent parasites often carried treatment-emerging mutations. Cipargamin will be developed further with an appropriate combo partner (Schmitt et al., 2022[97]).

### Artefenomel

Synonym: OZ439

**Figure 8:**
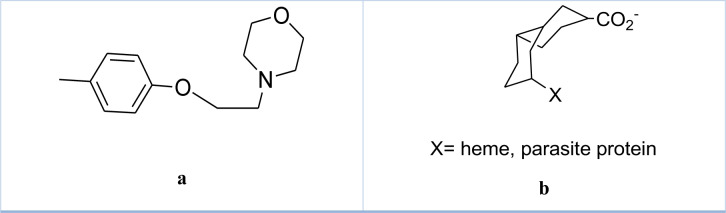
(a) Artefenomel, (b) parasite proteins

Artefenomel is a novel synthetic trioxolane derivative with high antimalarial activity and the same pharmacophore as Artemisinin (Phyo et al., 2016[87]). Drug OZ439 Clinical Trials were registered under NCT02573857. Effects of Artefenomel on parasitemia were found to be exerted for up to sixteen days (Collins et al., 2022[23]). OZ439 shows minimum inhibitory concentration for more than one week after single administration. It is considered a single-dose cure treatment and is very helpful in combination therapy. Potent OZ439 (Figure 8(Fig. 8)) shows potent activity against early blood-stage *P. falciparum* malaria infection in healthy subjects. It is a synthetic ozonide with properties similar to that of Artemisinin. When used in combination therapy, Artefenomel shows high activity to fix parasitemia (McCarthy et al., 2016[66][67]).

When hemoglobin is digested, the peroxide bond in Artemisinin and Artefenomel is activated by reduction and releases the ferrous heme group, generating carbon-centered radicals that alkylate heme and parasite proteins (Jourdan et al., 2016[52]). The drug rapidly kills all erythrocytic asexual *Plasmodium falciparum* stages. The IC_50_ value was similar to other Artemisinin derivatives. The drug shows t_1/2 _of 3 hrs and a multiphase end point half-life of 25-30 hrs. Food shows no effect on the t_1/2 _of OZ439.

### Phase I

Medicines for the Malaria Venture (MMV) Switzerland carried out and provided financial support for the Comprehensive Phase One Miramar (3400 Business Way, Miramar, FL 33025, USA) Phase I clinical investigation. The analysis was conducted under a US Investigational New Drug Application (IND). Healthy male or female non-smokers with a known medical history and a physical exam like an ECG are chosen for clinical trials. The age of the subjects lies between 18 and 55 years with a body weight ≥ 60 kg. Subjects enrolled are given different doses. A total of 26 candidates are enrolled, of which 10 are enrolled in cohort 1, 8 are enrolled in cohort 2 and 8 candidates are enrolled in cohort 3. The mean ages of candidates were 35.6 years (range 21-55 years). 80.8 % of the candidates were men, all Hispanic or Latino.

### Study design

The drug was studied by dividing it into two parts - Part A and Part B. A single rising dose study was performed by taking oral doses of Artefenomel up to 50 mg to 1600 mg. The drug was investigated by dividing the doses among three cohorts. In Cohort 1, the dosage was given as capsules, and in Cohort 2, the drug oral dispersion phase was investigated. After studying the safety among two cohorts, Cohort 3 was added to study the oral dispersion of OZ439. Multiple rising doses were performed for Artefenomel (Table 12(Tab. 12)). It showed the different pharmacokinetic values for additional doses of OZ439. C_max_ for 50 gm was 17.4, t_max_ was 3.00, and AUC was 102. It varies with increasing doses of the drug. The food and pharmacokinetics value of doses 50 mg, 100 mg, 200 mg, 400 mg, 800 mg, and 1200 mg are shown in (Table 12(Tab. 12)).

The drug is well tolerated in fed or fasted in both conditions. Adverse effects experienced by the candidates were diarrhea in 3/6 candidates, nausea in 3/6 candidates, gastrointestinal hypermotility in 2/6 candidates, abdominal discomfort in 1/6 candidates who were given 1600 mg oral dispersion, dyspepsia in 1/17 candidates and headache in 1/8 candidates which are given 200 mg capsule, vasovagal syncope in 1/8 candidates which are given 800 mg capsule (Moehrle et al., 2013[69]). Adults from Thailand with *P. falciparum* and *P. vivax* malaria were evaluated for the safety and effectiveness of a single dosage of Artefenomel (Wicha et al., 2022[117]). In Southeast Asia, OZ439 is undergoing phase IIa clinical studies. OZ439 clears the parasite from RBC cells at the same rate as artemisinin. Artemisinin and OZ439 show the exact mechanism of action, and both have a peroxide bridge that breaks into free radicals in the parasite.

### Phase II

Thailand's Shoklo Malaria Research Unit and the Hospital for Tropical Diseases in Bangkok conducted open-label trials in which one oral dose of 200 mg, 400 mg, 800 mg, or 1200 mg was administered to adult subjects. In Table 13(Tab. 13) plasma pharmacokinetics variables of Artefenomel were shown.

Peak plasma concentration (C_max_), maximum plasma concentration (T_max_), and area under the concentration-time curve (AUC_0-t_) from zero hours to the last pharmacokinetic sample (96 hours) are all terms used for the drug. The terminal phase half-life is t_1/2_, and the area under the concentration-time curve from zero hours to infinity is AUC_0-∞_. From October 24, 2010, to May 25, 2012, the trial was conducted in which 82 patients were enrolled as 20 patients in each of 200 mg, 400 mg, and 800 mg cohorts. 21 patients were in 1200 mg cohort. *Plasmodium* *falciparum* reduction rates per 24 hours ranged from 0.90 to 1.88, whereas *Plasmodium vivax* reduction rates ranged from 2.09 to 2.053. Adult male and female patients aged 18-60 years and weighing 60-90 kg with 5000-50.000 parasites were evaluated. When used as a partner drug in combination therapy, the drug is well tolerated up to high doses of 1600 mg (Phyo et al., 2016[87]).

Further studies improve the pharmacodynamics and pharmacokinetics of artemisinins and lead to synthesizing the more potent compound OZ439. Improved pharmacokinetic properties of the OZ277 drug leads to the formation of a new chemical moiety, OZ439 (Lau et al., 2015[54]). OZ439-piperaquine combination therapy in phase IIb trials is identified based on safety and efficacy before employing on too older and younger children. In March, the third review by the independent Safety Monitoring Board on data allowed the use of drugs in children of the lowest age group (6 months to ≤ 2 years). Phase IIb trial started in 2015 and the decision was made to take it in phase III trials. The extended half-life peroxide antimalarial OZ439 (Artefenomel), a second-generation ozonide clinical candidate, has advanced stages of development when combined with ferroquine. It is the first to be evaluated clinically (Siddiqui et al., 2022[99]). OZ439 shows increased pharmacokinetic properties and prolonged blood concentrations and maintains the necessary concentration of the drug in the blood to kill parasites. OZ439 was 20 folds more stable than OZ277 (Susan et al., 2011[107]). 

OZ439 and OZ609 will be highly potent against kelch 13 mutant isolates like *P. falciparum* Cam3.IR539 T following drug pulses of at least 48 h because *in vitro *RSA predicts the potency of parasite compounds with increased tolerance to Artemisinin and its derivatives. This is consistent with modeling results and recent trial data indicating a 6-day Artemisinin-based combination therapy regimen with 97.7 % efficacy. However, it is evident that the situation on the ground needs to be constantly managed by testing ozonides against new clinical isolates and, most importantly, under clinically relevant conditions (Jamshidi et al., 2019[50]).

### Phase IIb 

The efficacy, safety, acceptability, and pharmacokinetics of a single-dose ferroquine and Artefenomel regimen in adults and children with uncomplicated *Plasmodium falciparum* malaria were examined in a randomized, double-blind, phase IIb research. With a progressive age step-down technique and a ferroqine dose step-up procedure, adults and children were progressively enrolled in 4 cohorts. The independent DMC reviewed the safety data of the first group (>14 years to >70 years) before recruiting successively younger patients. Using an Integrated Web Recognition System (IWRS), permuted block randomization schedules, three randomization lists, and eligible patients, the treatment arms of Artefenomel/ferroquine 800/400 mg, 800/600 mg, 800/900 mg, and 800/1200 mg were centrally randomized. Within Africa, age classes (>14 to 70 years, >5 to 14 years, >2 to 5 years, and >6 months to 2 years) were stratified along with regions, treatment administration and blinding techniques. Patients from Vietnam and six African nations, ranging in age from 6 months to 70 years, participated in the study, a randomized, double-masked, single-dose, multi-arm clinical trial. Three hours before and two hours after the administration, a healthcare professional gave patients exploring formulations of ferroquine and Artefenomel orally while fasting. Artefenomel suspension was administered later (unblinded) after patients received ferroquine capsules in a double-blinded manner, wherein patients in each weight band received the same number of capsules (6 or 8, depending on the dose). A solution was produced for children by opening ferroquine capsules. Depending on weight, the total administration volume for children aged 2 to 5 was between 110 and 180 mL. Ferroquine was not to be dosed again if patients vomited during or after receiving it but before receiving Artefenomel. Instead, the patients were given emergency care and were removed from the trial (but were followed up for safety). Redoses of Artefenomel were required for patients who vomited within 5 minutes of the drug administration. Patients who vomited within 5 minutes of starting Artefenomel administration were instructed to finish the remaining dose of Artefenomel but not to take another dose. To assess the drug's effectiveness, safety, and pharmacokinetics, patients were monitored for 63 days. The primary efficacy endpoint was the adequate clinical and parasitological response (ACPR), measured on Day 28 in the Per-Protocol [PP] Set using exclusively African patients aged 5 years and older. We looked at the prevalence of kelch-13 mutations and the exposure-response association for the PCR-adjusted ACPR at Day 28. In total, 373 individuals received care, including 289 African patients under the age of 5 (77.5 %), 64 African patients above the age of 5, and 20 Asian patients. The goal efficacy criterion for PCR-adjusted ACPR was not satisfied by any treatment groups (Adoke et al., 2021[3]).

### KAF156 

New antimalarial compounds in advanced phases of development include ganaplacide (KAF156), which shows promising results by inhibiting *P. falciparum* gametocytogenesis and oocyst development in Anopheles mosquitoes at the asexual blood stage. A synonym for KAF156 is GNF156/Ganaplacide (Yipsirimetee et al., 2022[120]). KAF156 represents novel antimalarial drug classes presently being investigated in extended phase II and phase III investigations. With action against the pre-erythrocytic liver stage, asexual and sexual blood stages, KAF156 is a novel imidazolopiperazine anti-malarial. Both KAF156 and piperaquine (PPQ) are CYP3A4 substrates and inhibitors; a two-way pharmacokinetic interaction was predicted based on *in vitro* evidence. Potential combined effects on the QT interval were also assessed. In November 2013, Novartis announced the discovery of a new antimalarial. In 2014, the drug is in phase II clinical trials. KAF156 can treat and prevent malaria by targeting both blood and liver infections. The drug attacks the parasites at both stages of their reproductive life cycle (Held et al., 2014[46]). The compounds were found and recognized through phenotypic screenings against asexual blood stages. Ganaplacid is an imidazole piperazine that inhibits several stages of the life cycle of *P. falciparum *(liver stage as well as asexual and sexual blood stages) and is in the nanomolar range. In preclinical and clinical trials, action against *P. falciparum* and *Plasmodium vivax* has been proven, and transmission-blocking activity has been demonstrated both *in vitro* and *in vivo*. The controlled human malaria infection (CHMI) paradigm demonstrates the causal preventive efficacy of ganaplacide. GNF179, a similar imidazole piperazine molecule, expands the endoplasmic reticulum, prevents the development of new permeability routes, and inhibits protein transport (Yipsirimetee et al., 2022[120]). 

**Figure 9:**
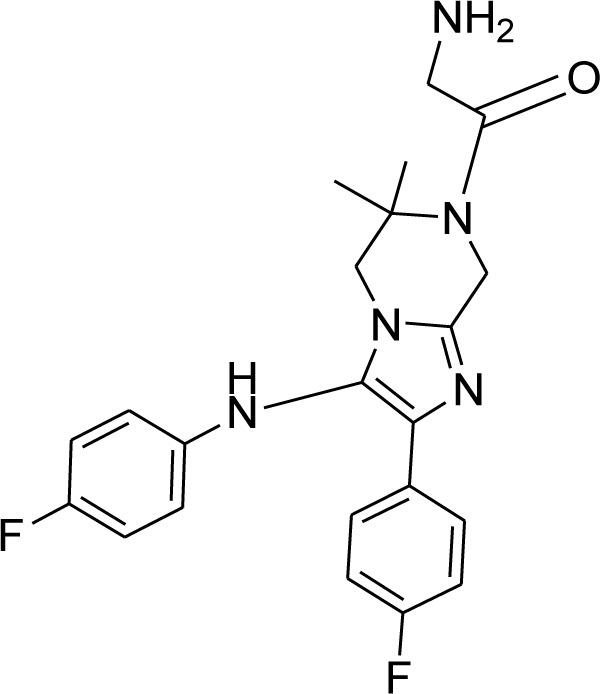
KAF156

Implementing a transmission-blocking strategy would be crucial for efforts to eliminate and subsequently eradicate malaria to be successful. Eliminating malaria is a problematic aim, so it's critical to stop the spread of Plasmodium parasites from person to person and from mosquito to person (Moyo et al., 2020[71]). The discovery of novel therapeutic strategies is crucial in the fight against the rise of Artemisinin-resistant parasites. Despite discovering new antimalarial drugs, these drugs are still in various phases of clinical development. When combined with the companion medication lumefantrine (NCT 04546633), one such medication, KAF-156 (Figure 9(Fig. 9)), is effective against Artemisinin-resistant parasites in a small adult trial (Jagannathan and Kakuru, 2022[48]).

With the help of Medicines for Malaria Venture (MMV), the Swiss Pharma Novartis will start the Phase IIb clinical trials to develop the next-generation antimalarial KAF156. The drug is known to fight against multidrug-resistant strains. KAF156 is an imidazol-piperazine compound which can be easily used as an oral tablet. KAF156 is found to be a superior drug to the other drugs as it targets the multiple stages. It also fights against the significant malaria pathogens, *P. falciparum* and *P.vivax* (Moyo et al., 2020[71]). 

Novartis joined forces with more than 80 leading international pharmaceutical and biotechnology companies in January 2016 to call for a global, unified front with governments against the rise of drug-resistant infections. With assistance from Medicines from Malaria Venture's research and funding, Novartis, in collaboration with the Bill and Melinda Gates Foundation, will take the lead in manufacturing the antimalarial drug KAF156. Novartis announced their cooperation and agreement to develop KAF156 and expand patient access on June 15, 2016. A collaborative research project with the Novartis Institute for Tropical Diseases, the Novartis Research Foundation Genomics Institute, and the Swiss Tropical and Public Health Institute is testing KAF 156 in Phase IIb clinical trials. This project is funded by the Wellcome Trust, MMV, and the Singapore Economic Development Board (Gupta et al., 2022[43]).

### Phase I 

An open-label, randomized, single-dose, parallel-group, non-confirmatory investigation was conducted on healthy participants. It took place at Melbourne, Australia's Nucleus Network. The main goal was to look into the possibility of pharmacokinetic interactions between KAF156 and piperaquine in healthy persons. Investigations into the safety and tolerability of KAF156 and piperaquine alone and when administered together in healthy participants, as well as possible effects on electrocardiogram (ECG) intervals (QT, PR, QRS) when KAF156 and piperaquine were given alone and in combination, were secondary goals. Males aged between 18 and 45 who were in good health were included in the study population. This was developed by screening medical history, physical examination, vital signs, ECG, and laboratory tests. Body mass index (BMI), which ranges from 18 to 30 kg/m^2^, was required for subjects to weigh at least 50 kg to participate in the study. Although female participants without the potential to become parents were welcome, none were chosen.

For KAF156 and piperaquine, the recommended doses were 800-1280 mg. Eight tablets of 100 mg strength were used to administer the 800 mg (KAF156 base equivalent) dose of KAF156. Tetraphosphate tetrahydrate was used to deliver piperaquine. 4 mg strength tablets containing the piperaquine dose of 1280 mg (piperaquine tetraphosphate equivalent) were administered. The trial included a screening phase of up to 26 days (Day 28 to Day 3), a baseline on Day 1, a single dosage therapy in 3 parallel treatment arms on Day 1, and a study completion evaluation. Each individual took roughly 61 days to complete the research, including the baseline phase without screening (Leong et al., 2018[57]).

### Phase II trials

A phase II open-label trial was carried out in Thailand and Vietnam to evaluate the antimalarial activity and efficacy of the drug. The study was conducted between March and August 2013. Patients were treated with 400 mg of the drug for a once-daily dose regimen for three days, followed by an independent cohort of 800 mg dose regimen, and responses were collected from cohorts up to 28 days. The median parasite clearance time with *P. falciparum* was 45 hrs, and with *P. vivax* was 24 hrs. Patients are sourced from the Bangkok Tropical Disease Hospital, the Hanoi National Institute of Malariology, Parasitology, and Entomology, the Shoklo Malaria Research Unit, and Mae Ramat Hospital along Thailand's northern border, the Plushing Hospital in eastern Thailand, and the Shoklo Malaria Research Unit. The eligible patients ranged in age from 20 to 60 years, had a body mass index of 40 to 90 kg, and had a current or prior fever, and infections with *P. vivax* and *P. falciparum* that were microscopically confirmed. Patients were divided into two cohorts: Cohort 1 with uncomplicated malaria was treated with 400 mg of KAF156, and Cohort 2 was treated with 800 mg of the drug. To evaluate the cure rate at 28 days and the possibility of a single-dosage cure, Cohort 3 was separated into two cohorts to treat patients with an 800 mg dose. 10 patients in each cohort are infected with *P. falciparum* in cohort 1 and *P. vivax* in cohort 2, respectively. One reinfection and seven recrudescent infections occurred among the 21 patients in Cohort 3 who received a single dosage and were monitored for 28 days. The dose regimen included 21 participants (11 with* P. falciparum *and 10 with *P. vivax)* (White et al., 2016[113]).

### Pharmacokinetic parameters of KAF156

On days 1 and 3, it took about 3 hours from the time KAF156 was administered for the plasma to reach its maximal plasma concentration (C_max_), with a cumulative terminal mean (± SD) of 44.1 ± 8.9 hours until the drug was eliminated entirely. The pharmacokinetic variables of KAF156 were AUC, C_max_, T_max_, Median, Terminal elimination half-life, and Accumulation ratio (Table 14(Tab. 14)). 

Of the patients in cohort 1, one did not have pharmacokinetic data. The pharmacokinetic characteristics of vomiting were not analyzed within three hours of dosage administration on four participants in the single-dose cohort. The plasma concentration-time curve from time 0 to time 24 is represented by the area under the curve (AUC_0-24_). AUC_0_-_last_ is the area under the plasma concentration-time curve from time 0 to the last measurable concentration. At the same time, AUC_0-inf_ is the area under the plasma concentration-time curve from time 0 to infinity, the observed maximum plasma concentration following drug administration, the CV coefficient of variation, the T_max _the time between drug delivery and the C_max_, and the VZ /F the apparent volume of distribution following oral dose. The accumulation ratio is AUC_0-24_ on Day 3 divided by AUC_0-24_ on Day 1.

### Clearance of parasites and fever

After receiving KAF156, the median time for the removal of fever was 14 hours (with a range of 4 to 30 hours) for 9 patients with *P. vivax* malaria, 6 hours (with a range of 4 to 24 hours) for multiple cohorts, and 4 hours for single dose cohorts for *P. falciparum* malaria. 1 patient with re-infection and 7 patients with recrudescent infections were found among the 21 *P. falciparum* malaria patients were given an 800 mg dose and monitored for 28 days. Gametocytemia was noted at baseline in two individuals with *P. vivax* malaria and disappeared in both patients up to 16 hours after receiving KAF156.

One *P. falciparum* malaria patient had intermittent gametocytemia from baseline to 54 hours after the dose was administered, and another patient had gametocytemia from baseline. Moreover, after therapy, 2 patients developed gametocytemia, with 1 having a single positive 24-hour reading and the other having positive 12- and 96-hour readings (16 gametocytes per cubic millimeter at the last reading). At this point, sampling stopped (White et al., 2016[113]). KAF156 offers a revolutionary drug that might be used to end malaria (Diagana, 2015[30]). Table 15(Tab. 15) shows the parasite clearance rates for patients with *Plasmodium vivax* and *Plasmodium falciparum* malaria who received several 400 mg of KAF 156 or a single 800 mg dosage.* Plasmodium vivax* and *Plasmodium falciparum* malaria patients received multiple 400 mg doses or a single 800 mg dosage of KAF156 to study parasite clearance.

The parasite clearance time of KAF156 is slightly slower than that of KAE609 (spiroindolone) treatment. *Falciparum* malaria is typically incurable with a single dosage of the rapidly removed antimalarial drug (terminal elimination half-life, 3 days); however, a 28-day parasite clearance adjusted cure rate of 67 % following a single dose of KAF156 suggests clinically significant *in vivo* potency (White et al., 2016[113]).

### DSM265

Chemical Formula: C_14_H_12_F_7_N _5s _

**Figure 10:**
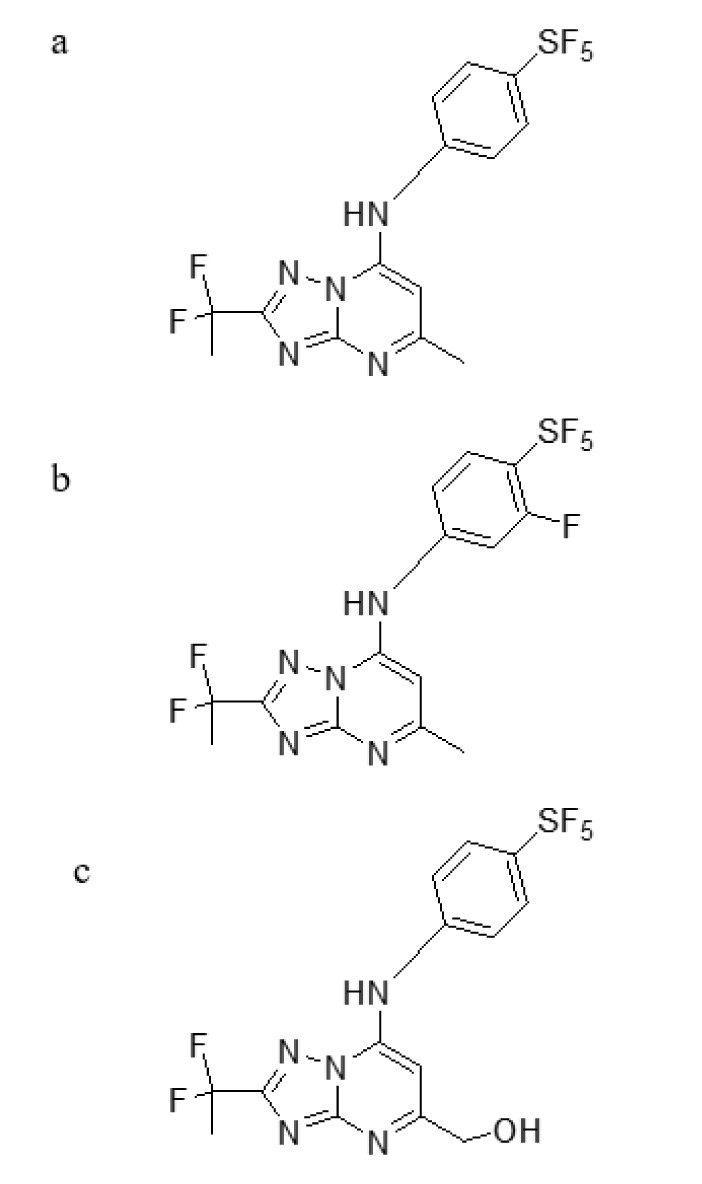
(a) Structure DSM265, (b) Structure DSM430, (c) Structure DSM450

DSM265 is 2-(1,1-difluoroethyl)-5-methyl-N-(4-(pentafluoro-16-sulfanyl)phenyl)-[1,2,4] triazolo [1,5-a] pyrimidin-7-amine. Combinations are active against all illness symptoms in a broad spectrum. The first DHODH inhibitor to reach clinical development for the treatment of malaria is DSM265, which is based on the triazolopyrimidine biosynthesis enzyme. DSM265 was developed by assessing its biological, pharmacological and pharmacokinetic characteristics. *Plasmodium falciparum's* DHODH is highly selective for DSM265 and effective against both its blood and liver stages as well as drug-resistant parasite isolates (Phillips et al., 2015[85]). DSM265 is a lipophilic compound with low aqueous solubility. Tisnerat et al. (2022[108]) identified it as an pyrimidine biosynthesis enzyme dihydroorotate dehydrogenase inhibitor based on triazolopyrimidines (DHODH). DSM265 (Figure 10(Fig. 10)) is a new drug to be used in combination therapy as a partner drug for either single-dose treatment or once-weekly treatment. It is a potent inhibitor of the *Plasmodium* enzyme. It shows good inhibitory activity against PfDHODH and* P. vivax *DHODH (PvDHODH) (Chowdhary et al., 2022[21]).

**Figure 11:**
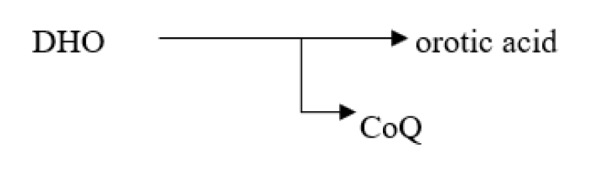
Oxidation of dihydroorotate

DSM430 is the main impurity in the DSM265, whereas DSM450 is the major metabolite of DSM265. DHODH is a mitochondrial enzyme that catalyzes the oxidation of dihydroorotate (DHO) to orotic acid (Figure 11(Fig. 11)).

It is a two-step reaction in the presence of coenzyme Q (CoQ) for reoxidation of FMN. DSM265 shows equal activity against all strains of *P. falciparum*. It is used against stage parasites and atovaquone compared to DSM265, DSM430, and DSM450. The DSM265 kill rate was slower than that of Artemisinin and chloroquine and similar to Atovaquone. Takeda Pharmaceuticals and Medicines for Malaria Venture propose proof of concept studies (Phase IIa) for patients and proof of concept studies (Phase Ib) to work together on molecular development. For both acute care and prophylaxis, DSM265 has potential as an antimalarial. The initials DSM stand for "Dallas-Seattle-Melbourne," the three locations where the principal researchers reside. These researchers tried hundreds of molecules before settling on the 265^th^ variant of the new drug, which is the most successful at blocking the parasite. DSM265 prevents the malaria parasite from replicating and invading hosts. The substance eradicates the parasite at every stage, from tiny spores ingested by the body through mosquito bites to the liver stage, when the larvae are fed and ultimately make their way into the bloodstream. DSM265 demonstrated its ability to protect and treat in a single dose. Peru has conducted studies on the drug's safety and effectiveness in collaboration with Takeda Pharmaceutical Company and the Global Health Innovative Technology (GHIT) fund. It was first found as part of a team effort led by Professor Margaret Phillips at the UT Southwestern Medical Center and supported by the National Institute of Health (NIH), MMV, and Wellcome Trust (Phillips et al., 2015[85]). It targets the ability of drug-resistant malaria parasites in the blood and liver to reproduce and kill them. For usage in combination therapy or to create a once-weekly preventative for people traveling to malaria-endemic areas, DSM265 would be paired with another drug. In Dr. Phillips' lab, work on DSM265 was discovered in 2008. Her group found the DHODH enzyme inhibitor essential for parasite survival (Mayoka et al., 2022[64]). A minimum set of two cohorts (20 patients) and a maximum set of six cohorts were used for the phase II open-label research of the medication (60 patients). For both *P.* *falciparum *and *P. vivax*, the initial dosage was 400 mg. Phase II intervention measures are to be found in detail in Table 16(Tab. 16).

**Figure 12:**
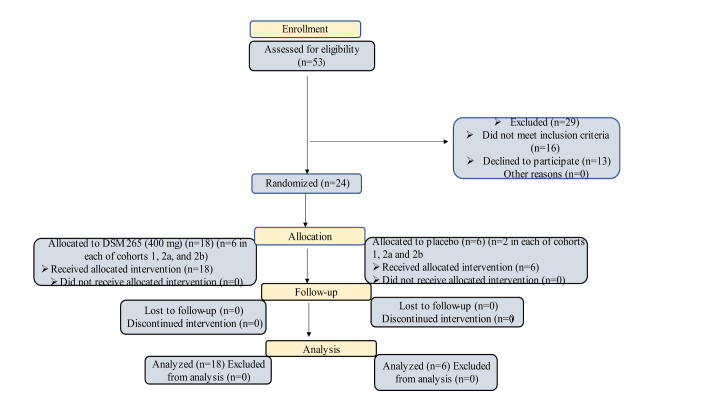
Study population allocation

Three cohorts of human volunteers participated in the single-center, double-blinded, randomized, placebo-controlled phase 1 study. The study was conducted between February 2016 and May 2017, with in-person procedures from April to September 2016. 24 of the 53 screened people were eligible, and groups of 8 participants were divided into cohorts 1, 2a, or 2b and given either DSM265 or a placebo (Figure 12(Fig. 12)). Participants comprised about 50 % of the population, and the average age ranged from 20 to 37 years. All cohorts and treatment arms shared similar baseline demographic traits (Table 15(Tab. 15)). All participants who underwent all in-person procedures, including CHMI, DSM265 dosage, and placebo dosing, were considered for the analysis. All placebo recipients and 4 of 6 DSM265 recipients in each cohort needed treatment by day 28. When all cohorts were combined, or even within cohorts, differences in treatment rates were not statistically significant (P >.28). It took substantially longer for DSM265 recipients than for placebo recipients to reach a parasite density of approximately 250 parasites/mL after the challenge (P =.004 for each group; P.001 overall. When comparing Cohort 1 (20.9 days; range, 12.4-23.4 days; to Cohort 2a (median, 15.3 days; range, 12.3-15.4 days; in which DSM265 was administered 7 days before challenge) and placebo recipients (8.3 days), Cohort 1 had the longest median time to reach a parasite density of less than 250 parasites/mL. Similar results were seen at the estimated RT-PCR limit of detection (20 parasites/mL): 67 % of DSM265-treated subjects and all placebo-recipient controls developed qRT-PCR positives, and the time to positivity was longer for DSM265 recipients compared to placebo recipients (P ≤ .02 for each cohort) (Murphy et al., 2018[73]).

Takeda Pharmaceuticals Company and MMV have advanced an antimalarial collaboration between (GHIT) Global Health Innovative Technology Fund, recently in Phase IIa clinical trials in Iquitos, Peru. The first antimalarial to target DHODH would be DSM265 if effective. The success of DSM265 in phase IIa clinical research is a significant step towards developing new or enhanced medications for treating and preventing malaria (Abd-Rahman et al., 2022[1]). In 2015 the compound entered a phase IIb trial in Iquitos, Peru. The parasite synthesizes DNA and RNA by nucleotide precursors, and the drug showed activity by targeting the ability of the parasite to synthesize. After a single dose in humans, DSM265 has been demonstrated to remain active for a very long time (Alzain et al., 2022[5]). One possible single-dose treatment for malaria is DSM265, which can also be taken in combination with other drugs.

The drug might also be developed as a once-weekly preventive therapy in preclinical animals, and the drug has demonstrated good tolerability and efficacy in preclinical animals. The drug activity can be shown as a one-dose combination therapy. The first clinical trial was studied in Australia, followed by an ongoing trial in Peru. Phase II clinical trials for malaria began in 2016, and Phase I clinical studies for malaria prevention (in volunteers) started in the USA in March 2016. DHODH is a mitochondrial enzyme required for pyrimidine biosynthesis. Pyrimidine uses coenzyme Q from mitochondria to catalyze the flavin-dependent oxidation of dihydroorotate to orotic acid (CoQ). An additional substrate was CoQ. Pyrimidine is essential because *Plasmodia *does not encode the pyridine salvage enzymes found in humans and other species, which are necessary for RNA and DNA creation. Pyrimidine and DHODH pathways are significant for the parasite.

In conjunction with MMV, numerous possible anti-malarial drugs are in various phases of research, including DSM265. MedicineNet compounds are urgently needed to fight malaria, as resistance to existing drugs is increasing. DHODH provides a critical function that could affect the parasite at several stages of its life cycle, including one elusive stage when it hides in the liver of the human host (Abd-Rahman et al., 2022[1]).

Participants and study design: A sequentially designed, open-label phase IIa proof-of-concept study was conducted at the Asociación Civil Selva Amazónica in Iquitos, Peru, to examine the efficacy of a single dose of DSM265 in treating adults with uncomplicated *P. falciparum* or *Plasmodium vivax* blood-stage malaria. In this area, *P. vivax* and *P. falciparum* are both endemic. Between January 12, 2015, and December 2, 2015, 45 Peruvian patients were sequentially enrolled. Of these, 24 had *P. falciparum* infection (cohort a), and 21 had *P. vivax* infection (cohort b).

For patients with *P. falciparum* malaria in the per-protocol population, 14 days after starting treatment, 11 (100 %) in the 400 mg group and eight (80 %) of 10 in the 250 mg group achieved ACPR. In the ITT research, ACPR was attained on day 14 by 11 (85 %) of 13 participants in the 400 mg group and eight (73 %) of 11 participants in the 250 mg group. The primary endpoint has not been reached for patients with *P. vivax* malaria.

According to the study methodology, on day 14, none of the four patients who took 400 mg, three (50 %) of the six who received 600 mg, and one (25 %) of the four who received 800 mg of DSM265 had a crude cure. In the ITT research, none of the five participants in the 400 mg group, three (33 %) of the nine in the 600 mg group, and one (14 %) of the seven in the 800 mg group had crude cure at day 14. A resistance-associated mutation was found in the gene encoding the DSM265 target DHODH in two of the four recurrent patients during the 28-day prolonged surveillance of *P. falciparum *patients. DSM265 has been highly accepted. The most typical side effects were pyrexia (20, or 44 % of the 45 patients) and headache (18, or 40 % of the 45 patients), both of which are symptoms of malaria. No patient experienced any severe adverse events or side effects that required cessation of the research due to therapy. The DHODH gene, missing at baseline, was changed in two parasite clones from the four patients with *P. falciparum* recurrence. Separate *in vitro* tests in earlier studies confirmed that the acquired mutations provided defence against DSM265 infection. This outcome highlights the need to use DSM265 to reduce the possibility of resistance in combination therapy. Monitoring the possible spread of resistance and any evidence of fitness costs associated with such mutations is essential. The WHO does not recommend any antimalarial drug for single-dose treatment of malaria. Hence, the promising efficacy of DSM265 against *P. falciparum *is of particular significance.

Also, proper randomization is avoided by the sequential research method. A limitation of this research was the minimal genetic diversity of the native *P. falciparum* parasites in Peru. This could lead to underestimating day-14 ACPR since reinfecting parasites can't be distinguished from the original infection when patients go home a few days after treatment. However, we presume that this confounding factor did not affect our findings. Another drawback is our patient population's low baseline parasitemia (1000-35000 parasites per μL) (Llanos-Cuentas et al., 2018[59]). The primary outcomes involved safety, pharmacokinetics, and pharmacodynamics (PD) related to a single 400 mg DSM265 dose. The non-compartmental study was used to calculate the PK parameters, which included the maximum plasma concentration (C_max_), the time point at which C_max_ was reached (T_max_), the removal half-life (t_1/2_) calculation, the area under the concentration-time curve from 0 h to the last time point calculation (AUC_0-last_), and the area under the concentration-time curve extrapolated from zero hours to infinity (AUC_0-∞_). The two PD variables of relevance in this investigation were the parasite reduction ratio (PRR) and the parasite clearance half-life. The parasite density ratio (PRR), which compares the parasite density at admission to the parasite density after 48 hours of antimalarial therapy, offers an estimate of the efficacy of the treatment.

The rate of gametocyte transmission to *Anopheles* mosquitoes and the impact of the second 400 mg DSM265 dose on gametocytemia were the secondary objectives. This study also aimed to describe the PK/PD connection of DSM265 in removing *P. falciparum* parasites. In the IBSM study, patients were given 150 mg of DSM265 and were combined with data from the most recent studies to do PK/PD modeling on the subjects (Collins et al., 2019[24]). 

The IBSM model was used in an open-label, phase Ib dose-finding study to describe the protective, PK, and PD effects of co-administration of Artefenomel and DSM265. The research was carried out from January to June 2015 at Q-Pharm Pty Ltd. (Australia, Brisbane). The study was open to safe men and women (of non-childbearing potential) between 18 and 55. A person was not eligible if he/she recently had systemic therapy with a drug that may have antimalarial action or an area where malaria was endemic for more than two weeks in the previous year. All subjects provided written, informed consent before their inclusion in the study.

This study was carried out per the Helsinki Declaration and was approved by the QIMR Berghofer Medical Research Institute's Human Research Ethics Committee (EC00278). The trial was listed on ClinicalTrials.gov with registration number NCT02389348 as of February 20, 2015.

The primary outcomes identified in the study protocol were the safety and PD related to Artefenomel and DSM265 coadministration. The study protocol's secondary objectives included the PK of OZ439 and DSM265 and the likelihood that a specific therapy would result in gametocytemia.

The maximum plasma concentration (C_max_), the time at which C_max_ was reached (T_max_), the half-life of elimination (t_1/2_), the apparent clearance (CL / F), and the apparent volume of distribution associated with the terminal elimination phase (Vz/F), where F is bioavailability, are all represented by the area under the concentration-time curve that extrapolates to infinity (AUC_0-∞_). The parasite reduction ratio (PRR) and the parasite clearance half-life were the PD variables of interest in this study (McCarthy et al., 2019[68]).

In collaboration with the Center for Infectious Disease Research (CID Research) and the University of Washington, the study was conducted at the Seattle Malaria Clinical Trials Center at the Fred Hutchinson Cancer Research Center (Seattle, WA). It is registered on Clinical Trials.gov (NCT02562872) and was approved by the Fred Hutchinson Cancer Research Center's Institutional Review Board. Adults (18-45 years old) in good health who never had malaria were enlisted. All cohorts used a preset randomization process that was only known to the research pharmacist to randomly assign participants to receive the drug or a placebo (DSM265 to placebo allocation ratio, 3:1). While sponsors, investigators, and research participants were kept blinded until the end of follow-up for the study, the safety review team was unblinded after day 60 during an interim database lock to allow efficacy and safety assessments.

### MMV390048

**Figure 13:**
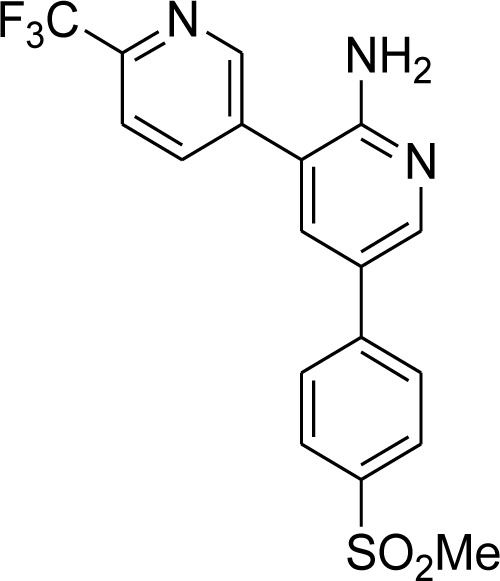
MMV390048

There are several antimalarial candidates either in development or already available. One of them is MMV390048 (Figure 13(Fig. 13)), a novel class of antimalarial chemotherapy that explicitly inhibits the *Plasmodium* phosphatidylinositol-4-kinase (PI4K), a protein necessary for all stages of the *Plasmodium* life cycle except hypnozoites. MMV390048 can also be referred to as MMV-048, MMV048 or MMV-390048. The chemical name of MMV390048 is 5-(4-(methyl sulfonyl phenyl)-6'-(trifluoromethyl)- [3,3-bipyridin]-2-amine (Demarta-Gatsi et al., 2022[27]).

The new compounds were identified by testing several millions of chemical samples against blood-stage human malaria caused by *P. falciparum* (Sonopo et al., 2016[104]). In September 2010, a compound code named MMV was called and displayed exceptional potency against the parasite. The compound is stable and shows significant activity when studied in *in vitro *experiments. In 2011, it was tested in animals, and the resultant data was more encouraging as MMV390048 showed a complete cure of infected animals when given orally at a low dose i.e. 20 mg/kg/dose. A molecule, code-named MMV390048, selected in 2012, was described by H3-D. The malaria parasite life cycle demonstrates robust behavior against many stages. It showed complex cures of animals infected with malaria parasites in a single cure when given orally. The drug was selected for further development based on the initial results of MMV390048. MMV390048 is an aminopyridine class drug that can potentially cure all strains of malaria in a single-dose cure. It also blocks the transmission of the parasite from person to person (MMV390048 shows potent activity against multiple points in the malaria parasite lifecycle). It competitively inhibited the binding of a single protein, P-14 kinase, in *Plasmodium falciparum (*Ghidelli-Disse et al., 2014[41]). The study will be conducted in a single center in two parallel cohorts with oral doses of MMV390048 as an open single-dose in healthy males and females. Healthy males and female subjects are between 18-55 years of age. Subjects will be screened within 28 days before entering the trial phase. 18 healthy males and females are included in this study; however, an additional cohort of subjects may also be added. In Phase I, the study was evaluated by administering the drug to healthy volunteers in a fast state. Each of the subjects receives one of the two MMV390048 prototype formulation doses. On Day 1, 40 mg drug with 240 ml of water was given. On Day 3, after 48 hrs post dose, they participate in other day therapy on Days 5, 7, 10, 14, 19, 26, and 29. The phase I intervention of MMV390048 is shown in Table 17(Tab. 17). 

Patients' eligibility as volunteers for Phase trials is based on age, gender, and health. The age of volunteers must lie between 18 years and 55 years. Both male and female are accepted for phase trial. For this study only healthy volunteers, including males or females of any race are included. Body weight at least 50 kg and a body mass 18-30 kg/m^2 ^are accepted for phase trials (Sinxadi et al., 2020[102]). The eligibility criteria for volunteers in phase trials are shown in Table 18(Tab. 18).

An optional cohort may be enrolled according to the adaptive features of the study. Up to 9 volunteers in this cohort will be given MMV390048 with milk or in the fed state (Abd-Rahman et al., 2022[1]). 

A double-blind, randomized, placebo-controlled, single ascending dosage study that was the first of its kind in humans was conducted. The antimalarial efficacy of MMV390048 was also studied in a voluntary infection investigation employing the *Plasmodium falciparum* mediated blood-stage malaria (IBSM) model.Research was conducted to decide on the MMV390048 tablet formulation for future investigations because the powder-in-bottle formulation employed in the first two trials had significant pharmacokinetic variability.

When given as a single oral dose of up to 120 mg, MMV390048 was generally well tolerated and had a quick absorption rate and a lengthy half-life. Although there was no conclusive link between MMV390048 exposure or dose in the first-in-human research, 12 adverse events were thought to be potentially related to it.

Even though the IBSM research revealed antimalarial efficacy, fast physical recovery happened in the majority of individuals following therapy with 20 mg of MMV390048, a level that is anticipated to be subtherapeutic. There was a significant reduction in the pharmacokinetic heterogeneity across participants as MMV390048 was reformulated into two tablet formulations (tartaric acid and syloid). Most research findings show that MMV390048 is well tolerated in people, and the pharmacokinetic characteristics imply that it may be utilized as a single-dose treatment or for prophylaxis of antimalarials. Adult Ethiopians with acute, simple *falciparum* or *vivax *malaria mono-infection are now being treated with MMV390048 in a phase IIa trial. The following registration numbers were used by the three clinical trials that are the subject of this discussion when they were submitted to ClinicalTrials.gov.: first-in-human study, NCT02230579; IBSM study, NCT02281344; and formulation optimization study, NCT02554799 (Sinxadi et al., 2020[102]).

### Ferroquine

A ferrocene-chloroquine derivative called ferroquine is effective against strains of *Plasmodium falciparum* that are resistant to the drug (Dubar et al., 2008[34]). The IUPAC name for ferroquine is (7-chloro-4-[(2-N, N-dimethyl aminomethyl) ferrocenylmethyl-amino]quinoline). Ferroquine can also be referred as SSR97193 (Pierrot et al., 2005[89]).

**Figure 14:**
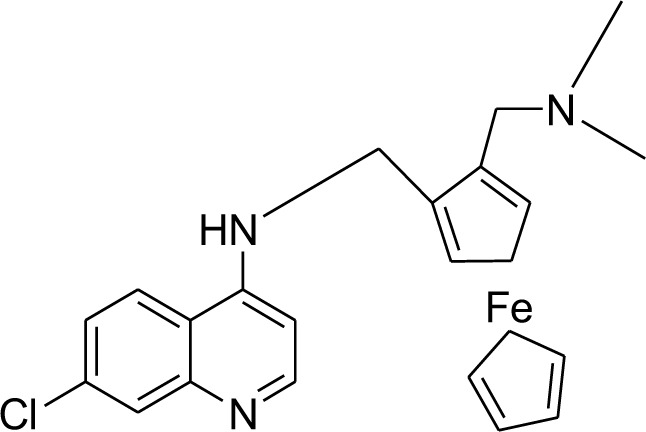
Ferroquine

Ferroquine (SSR97193) (Figure 14(Fig. 14)) is a new antimalarial drug candidate developed by Sanofi-Aventis (Chavain et al., 2008[18]), resulting from the incorporation of a metallocenic moiety to chloroquine (Daher et al., 2006[26]). As a single-dose drug for treating uncomplicated malaria, ferroquine was an antimalarial drug developed by Sanofi and Medicines for Malaria Venture (MMV) (Gansane et al., 2023[40]). Ferroquine mainly interacts with hemozoin surfaces and prevents parasite growth (Dubar et al., 2011[33]). To further understand the drug's pharmacokinetic (PK) and pharmacodynamic (PD) characteristics, volunteers were evaluated in an experimentally produced blood-stage malaria infection context (McCarthy et al., 2016[66][67]). Ferroquine was oxidized by CYP isoforms, i.e. 2C9, 2C19, 2D6 and 3A4. It has a very low hepatic metabolism rate (Daher et al., 2016[25]). FQ was expected to act by concentrating the digestive vacuole (DV) parasite and, thereby, preventing the development of hemozoin and eventually contributing to the parasite's death. The presence of the ferrocene center leads to solid solid anti-plasmodial action (Chavain et al., 2008[18]).

### Efficacy on clinical isolates 

Using *Plasmodium falciparum*-infected erythrocytes as a model, the chemicals were assessed based on the accepted approach for inhibiting parasite development. The growth inhibition rate varies with the country and the number of candidates. In Table 19(Tab. 19) different studies performed on clinical isolates were shown (Biot et al., 2011[15]).

### Phase I trials

335 participants or patients received FQ (SSR97193) as of June 28, 2010. Four investigations with asymptomatic *P. falciparum* patients in Africa (including adult African patients with *Plasmodium falciparum* mono-infection and parasitemia within the limits of 100 to 200,000 µl) contained 173 male subjects, as opposed to two research studies using healthy Caucasian participants. The initial and second cohorts, which included patients who were adults and kids who weighed more than 20 kg, have been finished (Biot et al., 2011[15]).

A placebo-controlled, randomized, single ascending dosage research was conducted to examine the antimalarial efficacy, pharmacokinetics, and protection of the Plasmodium phosphatidylinositol 4-kinase inhibitor in volunteers. In Part 1, there were 24 subjects (n = 8 per cohort, randomized 3:1 MMV390048: placebo), and in Part 2, there were 15 subjects (40 mg [n = 7] and 80 mg [n = 8]). One person was excluded from the study and removed from part 2 (80 mg group) prior to dosing. 

The rate of parasite clearance was higher in patients given 80 mg than it was in those given 40 mg (clearance half-life: 5.5 hours [95 % confidence interval: 5.2-6.0 hours] vs 6.4 hours [95 % CI: 6.0-6.9 hours]; P =.005).

A single 120 mg dose was predicted by pharmacokinetic/pharmacodynamic modeling to produce a sufficient clinical and parasitological response with 92 % certainty. Similarly, a minimum inhibitory concentration of 83 µg/mL and a minimum parasiticidal concentration were predicted to produce 90 % of the maximum effect of 238 µg/mL (McCarthy et al., 2019[68]). 

### Phase II trials

The single-centered clinical trial was performed in phase II. The screening included men and non-pregnant women aged 18-50 years. A single 800 mg of ferroquine was administered after the subjects received an inoculation of human erythrocytes infected with 1800 live *Plasmodium falciparum* 3D7A. Between October 17 and December 11, 2013, it was carried out at Q Pharm Pty Ltd in Australia. 

The primary goal of the phase investigations was to determine the pharmacokinetics and clearance rate of the *P. falciparum* parasite from the volunteers' blood (Mayoka et al., 2022[64]). The individuals were exposed to about 1800 live *Plasmodium falciparum* 3D7A inoculations intravenously. A single 100 mg capsule containing 800 mg of ferroquine was given to fasting volunteers. The effects of parasitemia on patients were observed for up to 16 days. Blood samples were taken at 0, 1, 2, 4, 6, 8, 12, 24, 48, 72, 96, and 144 hours after the ferroquine dose, as well as on days 8, 11, 14 and 28 after the trial, to determine the concentration of ferroquine in whole blood. Blood samples were taken at 0, 1, 2, 4, 6, 8, 12, 24, 48, 72, 96, and 144 hours after the ferroquine dose, as well as on days 8, 11, 14 and 28 after the trial, to determine the concentration of ferroquine in whole blood. Blood samples were taken at 0, 1, 2, 4, 6, 8, 12, 24, 48, 72, 96, and 144 hours after the ferroquine dose, as well as on days 8, 11, 14 and 28 after the trial, to determine the concentration of ferroquine in whole blood. 19 subjects were screened for this study, and eight were enrolled for further analysis. 

8 *Plasmodium falciparum*-infected participants were treated with 800 mg of ferroquine after their blood levels reached 1000 parasites per milliliter. These patients consisted of three men and five women, with a mean age of 26 (6.4) and a body mass index of 23.3 kg/m^2^ (±1.7). The non-compartment analysis parameters for determining pharmacokinetics have been shown in Table 20(Tab. 20).

The amount of time it takes for parasitemia to go down to an extrapolated intercept with either a 0.003 or 0.0002 parasites/mL threshold (equivalent to 1 parasite per 70 kg subject). Additional clinical trials will be necessary to confirm the safety profile of ferroquine further (McCarthy et al., 2016[67][68]).

## Discussion

A lot of work has been done on forming new anti-malarial drugs. The active substances in early stages of clinical development are Tafenoquine, an 8-amino quinoline com-pound, DSM265, a triazolopyrimidine derivative, KAF156, an imidazol-piperazine, KAE609, a spiroindo-lone derivative, OZ439, a trioxolane, and MMV390048. Due to its harmful effects, the ferroquine clinical development was completed in Phase III trials. Of these preclinical development drugs, only three display novel or known mechanisms of action. It is currently being done to support the beginning of human clinical trials to conduct additional safety and pharmacological preclinical evaluation. There are reasons for cautious optimism, such as the success of trials testing novel vaccine candidates and combinatorial interventions, the approval of the first malaria vaccine in history, and results from studies. To prioritize and fund the development of new therapeutic, preventative, and vaccine strategies against malaria, however, it is urgently necessary to close significant research gaps.

## Declaration of competing interest

The authors declare that they have no known competing financial interests or personal ties that could have seemed to affect the work provided in this study.

## Figures and Tables

**Table 1:**
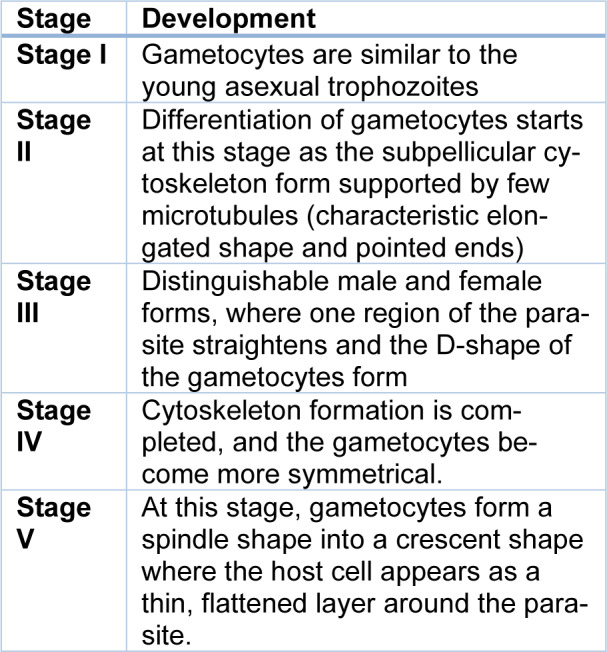
Five stages of gametocyte development

**Table 2:**
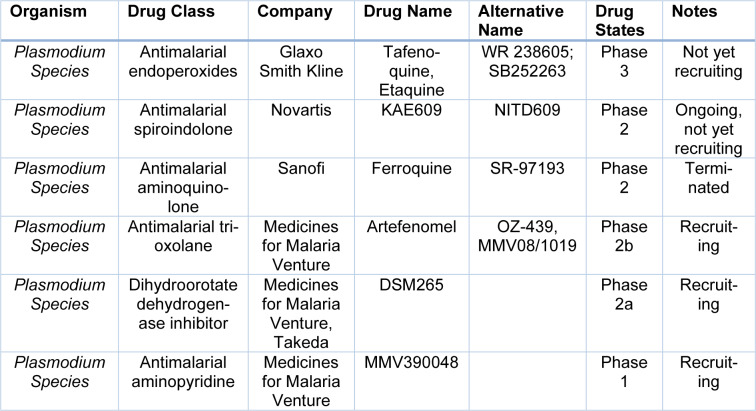
Protozoal drugs in development

**Table 3:**
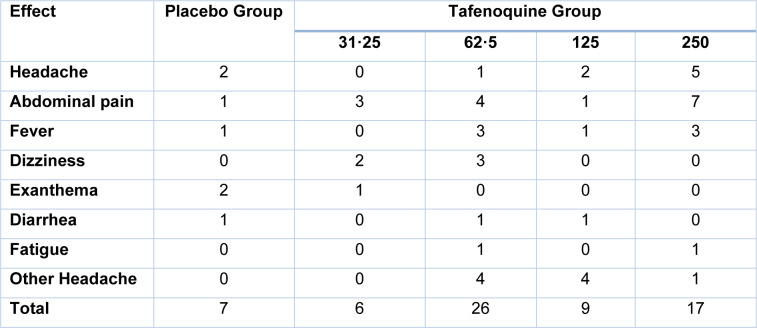
Adverse events reported by patients during 28 days of drug intake

**Table 4:**
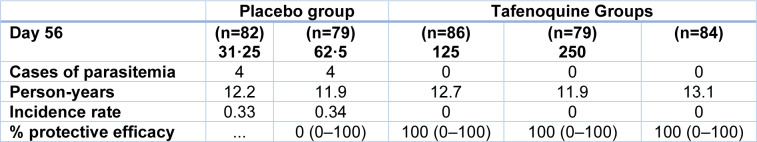
Results of effects of drug on day 56

**Table 5:**
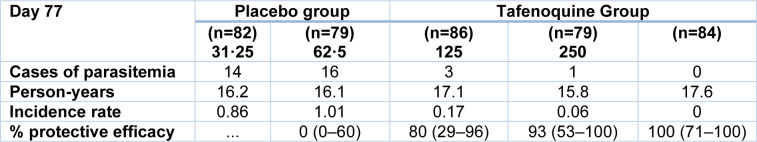
Results of drug dose on patients on day 77

**Table 6:**
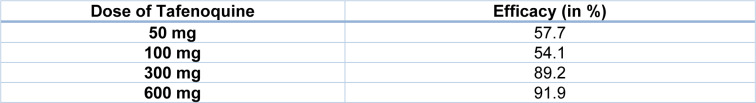
Relapse-free efficacy at 6 months of Tafenoquine

**Table 7:**
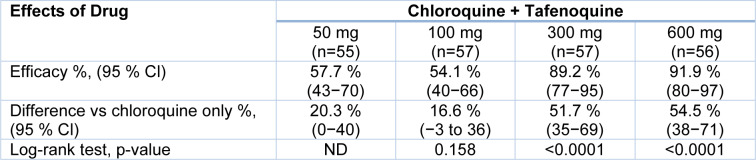
Relapse-free effectiveness estimates using Kaplan-Meier methods in the intention-to-treat population at 6 months after treatment

**Table 8:**
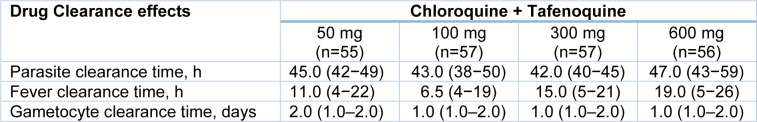
Median parasite, fever and gametocyte clearance times

**Table 9:**
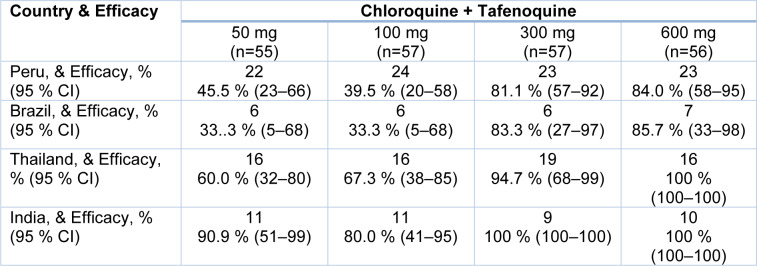
Kaplan-Meier estimates of relapse-free efficacy at 6 months after therapy by nation

**Table 10:**
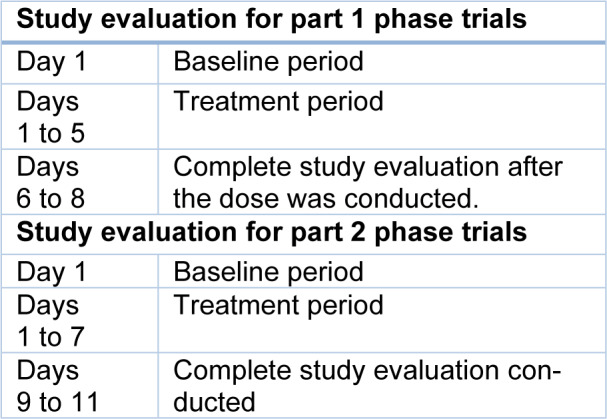
Evaluation of part 1 and part 2 phase trials

**Table 11:**
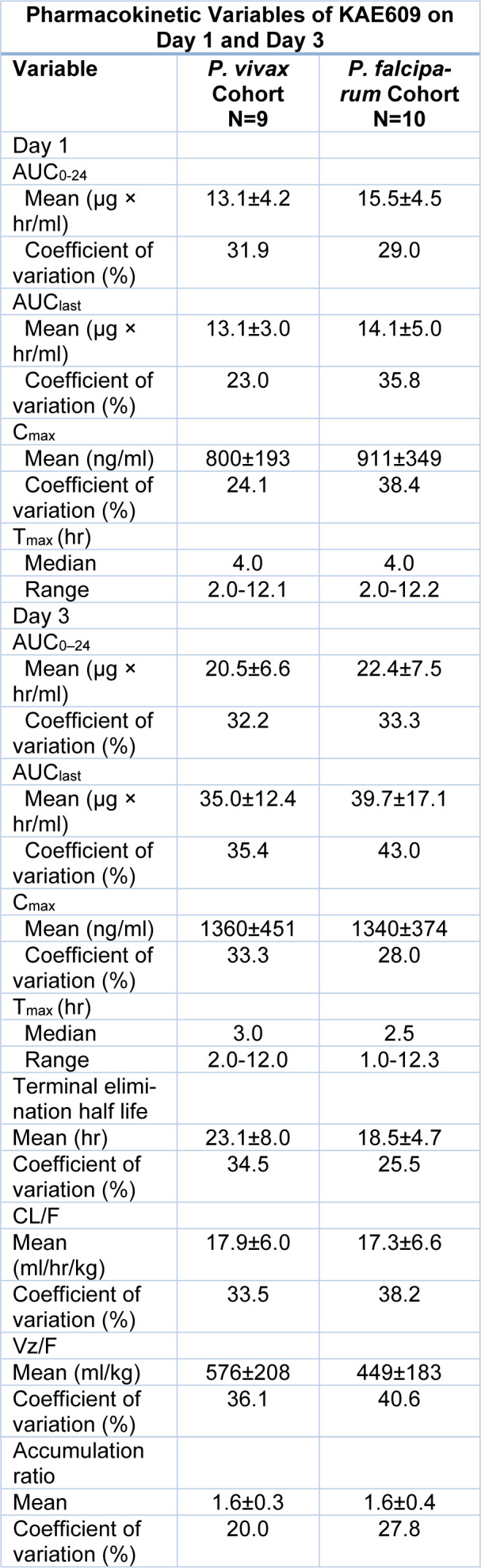
Results of pharmacokinetic variables of KAE609 for different cohorts

**Table 12:**
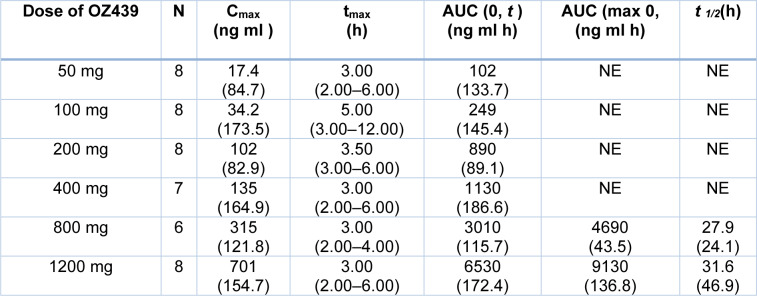
Results of food and pharmacokinetics value of Artefenomel

**Table 13:**
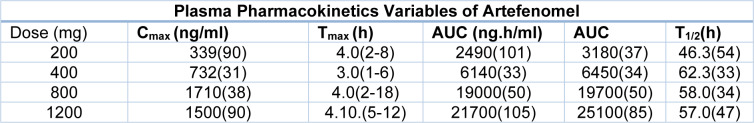
Plasma pharmacokinetics variables of Artefenomel

**Table 14:**
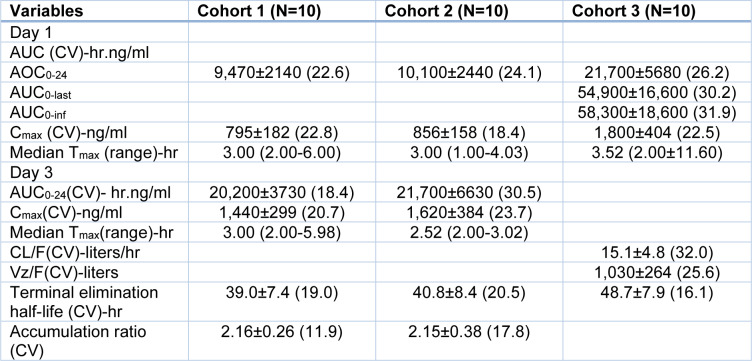
Pharmacokinetic variables of KAF156

**Table 15:**
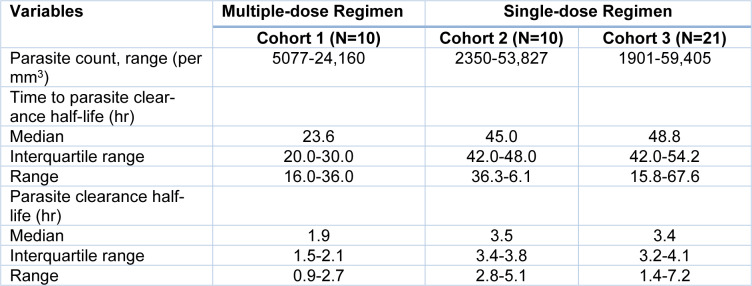
Patients who received multiple dosages showed parasite clearance

**Table 16:**
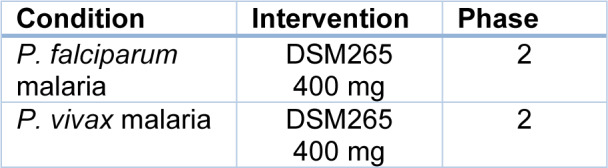
Intervention for malaria in phase II treated with* Plasmodium vivax *and *Plasmodium falciparum*

**Table 17:**
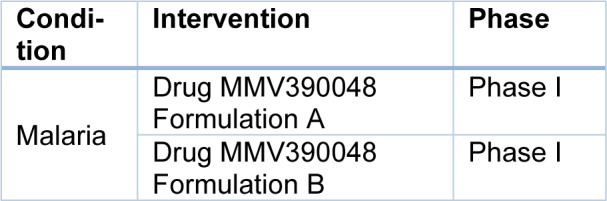
Intervention for drug MMV390048 under Phase I

**Table 18:**
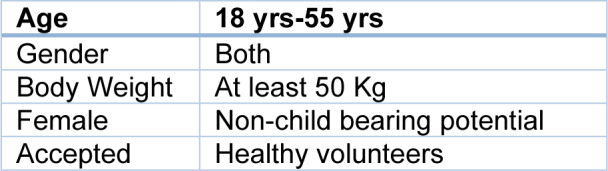
Eligibility criteria for Phase I trials

**Table 19:**
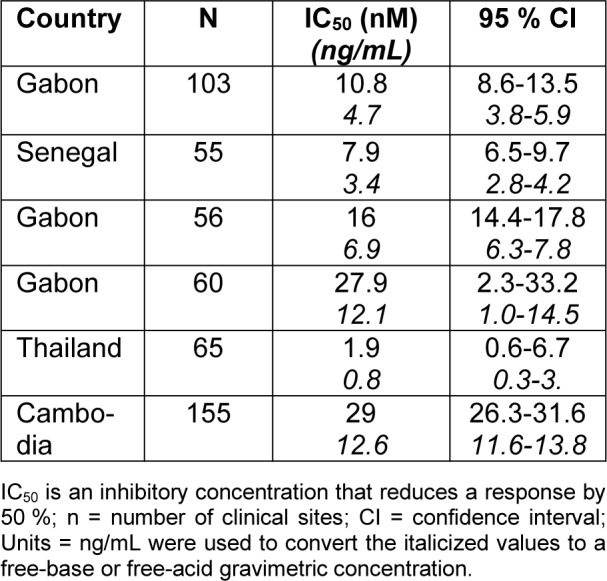
Impact of FQ (IC_50_ and 95 % confidence intervals for SSR97193 on *P.* *falciparum* clinical isolates from various studies)

**Table 20:**
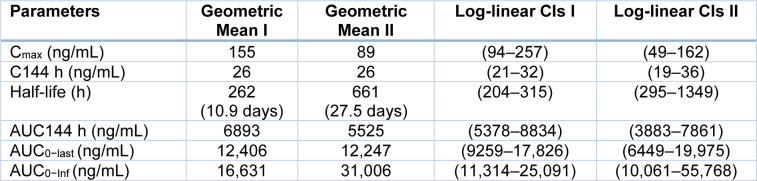
Non-compartment analysis parameter for pharmacokinetics determination

## References

[1] Abd-Rahman AN, Zaloumis S, McCarthy JS, Simpson JA, Commons RJ (2022). A scoping review of antimalarial drug candidates in phase I and II drug development. Antimicrob Agents Chemother.

[2] Abebaw A, Aschale Y, Kebede T, Hailu A (2022). The prevalence of symptomatic and asymptomatic malaria and its associated factors in Debre Elias district communities, northwest Ethiopia. Malar J.

[3] Adoke Y, Zoleko-Manego R, Ouoba S, Tiono AB, Kaguthi G, Bonzela JE (2021). A randomized, double-blind, phase 2b study to investigate the efficacy, safety, tolerability and pharmacokinetics of a single-dose regimen of ferroquine with Artefenomel in adults and children with uncomplicated Plasmodium falciparum malaria. Malar J.

[4] Aleshnick M, Florez-Cuadros M, Martinson T, Wilder BK (2022). Monoclonal antibodies for malaria prevention. Mol Ther.

[5] Alzain AA, Ahmed ZAM, Mahadi MA, khairy EA, Elbadwi FA (2022). Identification of novel Plasmodium falciparum dihydroorotate dehydrogenase inhibitors for malaria using in silico studies. Sci Afr.

[6] Amaratunga C, Lim P, Suon S, Sreng S, Mao S, Sopha C (2016). Dihydroartemisnin-piperaquine resistance in Plasmodium falciparum malaria in Cambodia: a multisite prospective cohort study. Lancet Infect Dis.

[7] Amina K, Giuliana G, Prato M (2010). From control to eradication of malaria: the end of being stuck in second gear?. Asian Pac J Trop Med.

[8] Angrisano F, Tan YH, Sturm A, McFadden GI, Baum J (2012). Malaria parasite colonisation of the mosquito midgut - placing the plasmodium ookinete centre stage. Int J Parasitol.

[9] Ariey F, Witkowski B, Amaratunga C, Beghain J, Langlois AC, Khim N (2014). A molecular marker of Artemisinin-resistant Plasmodium falciparum malaria. Nature.

[10] Ashley EA, Phyo AP (2018). Drugs in development for malaria. Drugs.

[11] Band G, Leffler EM, Jallow M, Sisay-Joof F, Ndila CM (2022). Malaria protection due to sickle haemoglobin depends on parasite genotype. Nature.

[12] Bantuchai S, Imad H, Nguitragool W (2022). Plasmodium vivax gametocytes and transmission. Parasitol Int.

[13] Bhattacharjee D, Shivaprakash G (2016). Drug resistance in malaria - in a nutshell. J App Pharm Sci.

[14] Bhuvaneshwari B, Kondaveti SS (2015). Pharmacology of newer antimalarial drugs: review article. J Evid Based Med Healthc.

[15] Biot C, Nosten F, Fraisse L, Ter-Minassian D, Khalife J, Dive D (2011). The antimalarial ferroquine: from bench to clinic. Parasite.

[16] Birkholtz LM, Alano P, Leroy D (2022). Transmission-blocking drugs for malaria elimination. Trends Parasitol.

[17] Charles BG, Miller AK, Nasveld PE, Reid MG, Harris IE, Edstein MD (2007). Population pharmacokinetics of Tafenoquine during malaria prophylaxis in healthy subjects. Antimicrob Agents Chemother.

[18] Chavain N, Vezin H, Dive D, Touati N, Paul JF, Buisine E (2008). Investigation of the redox behavior of ferroquine, a new antimalarial. Mol Pharm.

[19] Chilengi R, Juma R, Abdallah AM, Bashraheil M, Lodenyo H, Nyakundi P (2011). A phase I trial to evaluate the safety and pharmacokinetics of low-dose methotrexate as an anti-malarial drug in Kenyan adult healthy volunteers. Malar J.

[20] Chora ÂF, Mota MM, Prudêncio M (2022). The reciprocal influence of the liver and blood stages of the malaria parasite’s life cycle. Int J Parasitol.

[21] Chowdhary S, Shalini, Mosnier J, Fonta I, Pradines B, Cele N (2022). Synthesis, anti-plasmodial activities, and mechanistic insights of 4-aminoquinoline-triazolopyrimidine hybrids. ACS Med Chem Lett.

[22] Cohen MJ, Smith LD, Cotter C, Ward A, Yamey G (2012). Malaria resurgence: a systematic review and assessment of its causes. Malar J.

[23] Collins KA, Abd-Rahman AN, Marquart L, Ballard E, Gobeau N, Griffin P (2022). Antimalarial activity of artefenomel against asexual parasites and transmissible gametocytes during experimental blood-stage plasmodium Vivax infection. J Infect Dis.

[24] Collins KA, Rückle T, Elliott S, Marquart L, Ballard E, Chalon S (2019). DSM265 at 400 milligrams clears asexual stage parasites but not mature gametocytes from the blood of healthy subjects experimentally infected with Plasmodium falciparum. Antimicrob Agents Chemother.

[25] Daher W, Pelinski L, Klieber S, Sadoun F, Meunier V, Bourrie M (2016). In vitro metabolism of ferroquine (SSR97193) in animal and human hepatic models and antimalarial activity of major metabolites on Plasmodium Falciparum. Antimicrob Agents Chemother.

[26] Daher W, Pelinski L, Klieber S, Sadoun F, Meunier V, Bourrie M (2006). In vitro metabolism of ferroquine (SSR97193) in animal and human hepatic models and antimalarial activity of major metabolites on Plasmodium falciparum. Drug Metab Dispos.

[27] Demarta-Gatsi C, Donini C, Duffy J, Sadler C, Stewart J, Barber JA (2022). Malarial PI4K inhibitor induced diaphragmatic hernias in rat: potential link with mammalian kinase inhibition. Birth Defects Res.

[28] Dennis ASM, Lehane AM, Ridgway MC, Holleran JP, Kirk K (2018). Cell swelling induced by the antimalarial KAE609 (Cipargamin) and other PfATP4-associated antimalarials. Antimicrob Agents Chemother.

[29] Deshpande S, Kuppast B (2016). 4-Aminoquinolones: an overview of antimalarial chemotherapy. Med Chem.

[30] Diagana TT (2015). Supporting malaria elimination with 21st-century antimalarial drug. Drug Discov Today.

[31] Doerig C, Baker D, Billker O, Blackman MJ, Chitnis C, Dhar Kumar S (2009). Signalling in malaria parasites. The MALSIG consortium. Parasite.

[32] Dow GS, Smith BL (2022). A phase II, double blind, placebo-controlled, randomized evaluation of the safety and efficacy of Tafenoquine in patients with mild-moderate COVID-19 disease. New Microbes New Infect.

[33] Dubar F, Egan TJ, Pradines B, Kuter D, Ncokazi KK, Forge D (2011). The antimalarial ferroquine: role of the metal and intramolecular hydrogen bond in activity and resistance. ACS Chem Biol.

[34] Dubar F, Khalife J, Brocard J, Dive D, Biot C (2008). Ferroquine, an ingenious antimalarial drug –Thoughts on the mechanism of action. Molecules.

[35] Egwu CO, Pério P, Augereau JM, Tsamesidis I, Benoit-Vical F (2022). Resistance to artemisinin in falciparum malaria parasites: A redox-mediated phenomenon. Free Radic Biol Med.

[36] Flegg JA, Guerin PJ, White NJ, Stepniewska K (2011). Standardizing the measurement of parasite clearance in falciparum malaria: the parasite clearance estimator. Malar J.

[37] Foley M, Tilley L (1998). Quinoline antimalarials: mechanisms of action and resistance and prospects for new agents. Pharmacol Ther.

[38] Fujioka H, Aikawa M (2002). Structure and life cycle. Chem Immunol.

[39] Gabbiani C, Messori L, Cinellu MA, Casini A, Mura P, Sannella AR (2009). Outstanding plasmodicidal properties within a small panel of metallic compounds: hints for the development of new metal-based antimalarials. J Inorg Biochem.

[40] Gansane A, Lingani M, Yeka A, Nahum A, Bouyou-Akotet M, Mombo-Ngoma G (2023). Randomized, open-label, phase 2a study to evaluate the contribution of Artefenomel to the clinical and parasiticidal activity of Artefenomel plus ferroquine in African patients with uncomplicated Plasmodium falciparum malaria. Malar J.

[41] Ghidelli-Disse S, Lafuente-Monasterio MJ, Waterson D, Witty M, Younis Y, Paquet T (2014). Identification of Plasmodium PI4 kinase as target of MMV390048 by chemoproteomics. Malar J.

[42] Gupta S (2015). Mastering malaria: what helps and what hurts. Proc Natl Acad Sci U S A.

[43] Gupta Y, Sharma N, Singh S, Romero JG, Rajendran V, Mogire RM (2022). The multistage antimalarial compound Calxinin perturbates P. falciparum Ca2+ homeostasis by targeting a unique ion channel. Pharmaceutics.

[44] Hanhsen B, Farrukh A, Pradel G, Ngwa CJ (2022). The plasmodium falciparum CCCH zinc finger protein ZNF4 plays an important role in gametocyte exflagellation through the regulation of male enriched transcripts. Cells.

[45] Haston JC, Hwang J, Tan KR (2019). Guidance for using Tafenoquine for prevention and antirelapse therapy for malaria-United states, 2019. MMWR Morb Mortal Wkly Rep.

[46] Held J, Jeyaraj S, Kreidenweiss A (2014). Antimalarial compounds in Phase II clinical development. Expert Opin Investig Drugs.

[47] Huskey SE, Zhu CQ, Fredenhagen A, Kühnöl J, Luneau A, Jian Z (2016). KAE609 (Cipargamin), a new spiroindolone agent for the treatment of malaria: evaluation of the absorption, distribution, metabolism, and excretion of a Single Oral 300-mg Dose of (14C)KAE609 in healthy male subjects. Drug Metab Dispos.

[48] Jagannathan P, Kakuru A (2022). Malaria in 2022: increasing challenges, cautious optimism. Nat Commun.

[49] Jain DC, Bhakuni RS, Gupta MM, Sharma RP, Kahol AP, Dutta GP (2000). Domestication of Artemisia annua Plant and development of new antimalarial drugs arteether in India. J Sci Ind Res.

[50] Jamshidi E, Eftekhar Ardebili H, Yousefi-Nooraie R, Raeisi A, Malekafzali Ardakani H, Sadeghi R (2019). A social network analysis on immigrants and refugees access to services in the malaria elimination context. Malar J.

[51] Jean PLS, Xue Z, Carter N, Koh GCKW, Duparc S, Taylor M (2016). Tafenoquine treatment of Plasmodium vivax malaria: suggestive evidence that CYP2D6 reduced metabolism is not associated with relapse in the Phase 2b detective trial. Malar J.

[52] Jourdan J, Matile H, Reift E, Biehlmaier O, Dong Y, Wang X (2016). Monoclonal antibodies that recognize the alkylation signature of antimalarial ozonides OZ277 (arterolane) and OZ439 (Artefenomel). ACS Infect Dis.

[53] Lancet (2022). Malaria in 2022: a year of opportunity (Editorial). Lancet.

[54] Lau SH, Galván A, Merchant RR, Battilocchio C, Souto JA, Berry MB (2015). Machines vs malaria: A flow-based preparation of the drug candidate OZ439. Org Lett.

[55] Lee AH, Fidock DA (2016). Evidence of a mild mutator phenotype in Cambodian Plasmodium falciparum malaria parasites. PLOS One.

[56] Lell B, Faucher JF, Missinou MA, Borrmann S, Dangelmaier O, Horton J (2000). Malaria chemoprophylaxis with Tafenoquine: a randomised study. Lancet.

[57] Leong FJ, Jain JP, Feng Y, Goswami B, Stein DS (2018). A phase 1 evaluation of the pharmacokinetic/pharmacodynamic interaction of the anti-malarial agents KAF156 and piperaquine. Malar J.

[58] Leong FJ, Li R, Jain JP, Lefèvre G, Magnusson B, Diagana TT (2014). First-in-human randomized, double-blind, placebo-controlled, Single and multiple-ascending oral dose study of novel antimalarial spiroindolone KAE609 (Cipargamin) to assess its safety, tolerability, and pharmacokinetics in healthy adult volunteers. Antimicrob Agents Chemother.

[59] Llanos-Cuentas A, Casapia M, Chuquiyauri R, Hinojosa J, Kerr N, Rosario M (2018). Antimalarial activity of single-dose DSM265, a novel plasmodium dihydroorotate dehydrogenase inhibitor, in patients with uncomplicated Plasmodium falciparum or Plasmodium vivax malaria infection: a proof-of-concept, open-label, phase 2a study. Lancet Infect Dis.

[60] Llanos-Cuentas A, Lacerda MV, Rueangweerayut R, Krudsood S, Gupta SK, Kochar SK (2014). Tafenoquine plus chloroquine for the treatment and relapse prevention of Plasmodium vivax malaria (DETECTIVE): a multicentre, double-blind, randomised, phase 2b dose-selection study. Lancet.

[61] Llanos-Cuentas A, Manrrique P, Rosas-Aguirre A, Herrera S, Hsiang MS (2022). Tafenoquine for the treatment of Plasmodium vivax malaria. Expert Opin Pharmacother.

[62] Mahajan A, Kremer L, Louw S, Guérardel Y, Chibale K, Biot C (2011). Synthesis and in vitro antitubercular activity of ferrocene-based hydrazones. Bioorg Med Chem Lett.

[63] Marcos LA, Leung A, Kirkman L, Wormser GP (2022). Use of Tafenoquine to treat a patient with relapsing babesiosis with clinical and molecular evidence of resistance to azithromycin and atovaquone. IDCases.

[64] Mayoka G, Woodland JG, Chibale K (2022). Thwarting protein synthesis leads to malaria parasite paralysis. Trends Parasitol.

[65] McCarthy JS, Abd-Rahman AN, Collins KA, Marquart L, Griffin P, Kümmel A (2021). Defining the antimalarial activity of Cipargamin in healthy volunteers experimentally infected with blood-stage plasmodium falciparum. Antimicrob Agents Chemother.

[66] McCarthy JS, Baker M, O’Rourke P, Marquart L, Griffin P, Hooft van Huijsduijnen R (2016). Efficacy of OZ439 (Artefenomel) against early Plasmodium falciparum blood-stage malaria infection in healthy volunteers. J Antimicrob Chemother.

[67] McCarthy JS, Ruckle J, Djeriou E, Cantalloube C, TerMinassian D, Baker M (2016). A phase II pilot trial to evaluate safety and efficacy of ferroquine against early Plasmodium falciparum in an induced bloodstage malaria infection study. Malar J.

[68] McCarthy JS, Rückle T, Elliott SL, Ballard E, Collins KA, Marquart L (2019). A single-dose combination study with the experimental antimalarials Artefenomel and dsm265 to determine safety and antimalarial activity against blood-stage plasmodium falciparum in healthy volunteers. Antimicrob Agents Chemother.

[69] Moehrle JJ, Duparc S, Siethoff C, Giersbergen PLMV, Craft JC, Barnes SA (2013). First-in-man safety and pharmacokinetics of synthetic ozonide OZ439 demonstrates an improved exposure profile relative to other peroxide antimalarials. Br J Clin Pharmacol.

[70] Monroe A, Williams NA, Ogoma S, Karema C, Okumu F (2022). Reflections on the 2021 World Malaria Report and the future of malaria control. Malar J.

[71] Moyo P, Mugumbate G, Eloff JN, Louw AI, Maharaj VJ, Birkholtz LM (2020). Natural products: A potential source of malaria transmission blocking drugs?. Pharmaceuticals (Basel).

[72] Mukherjee P, Pradhan A, Shah F, Tekwani BL, Avery MA (2008). Structural insights into the Plasmodium falciparum histone deacetylase 1 (Pf HDAC-1): A novel target for the development of antimalarial therapy. Bioorg Med Chem.

[73] Murphy SC, Duke ER, Shipman KJ, Jensen RL, Fong Y, Ferguson S (2018). A randomized trial evaluating the prophylactic activity of DSM265 against preerythrocytic Plasmodium falciparum infection during controlled human malarial infection by mosquito bites and direct venous inoculation. J Infect Dis.

[74] Mushtaque Md, Shahjahan (2015). Reemergence of chloroquine (CQ) analogs as multi-targeting antimalarial agents: a review. Eur J Med Chem.

[75] Nandal R, Deep A, Singh I, Kaushik M, S (2019). L. H, Narasimhan B, et al. Synthesis of metal complexes of primaquine and in-vitro antimalarial evaluation against Plasmodium falciparum. Curr Bioact Compd.

[76] Nasveld PE, Edstein MD, Reid M, Brennan L, Harris IE, Kitchener SJ (2010). Randomized, double blind study of the safety, tolerability, and efficacy of tafenoquine versus mefloquine for malaria prophylaxis in nonimmune subjects. Antimicrob Agents Chemother.

[77] Styka AN, Savitz DA, National Academies of Sciences, Engineering, and Medicine, Health and Medicine Division, Committee to Review Long-Term Health Effects of Antimalarial Drugs, Board on Population Health and Public Health Practice (2020). Assessment of long-term health effects of antimalarial drugs when used for prophylaxis.

[78] Navarro M, Castro W, Martínez A, Sánchez Delgado RAS (2011). The mechanism of antimalarial action of (Au(CQ)(PPh3))PF6: structural effects and increased drug lipophilicity enhance heme aggregation inhibition at lipid/water interfaces. J Inorg Biochem.

[79] Neal AT, Schall JJ (2014). Life history focus on a malaria parasite: linked traits and variation among genetic clones. Evol Ecol.

[80] Noedl H, Se Y, Schaecher K, Smith BL, Socheat D, Fukuda MM (2008). Evidence of Artemisinin-resistant malaria in Western Cambodia. N Engl J Med.

[81] Nureye D, Assefa S (2020). Old and recent advances in life cycle, pathogenesis, diagnosis, prevention, and treatment of malaria including perspectives in Ethiopia. Sci World J.

[82] Paredes CF, Jose I, Santos-Preciado JI (2006). Problem pathogens: prevention of malaria in travellers. Lancet Infect Dis.

[83] Patarroyo ME, Cifuentes G, Rodríguez R (2008). Structural characterisation of sporozoite components for a multistage, multi-epitope, anti -malarial vaccine. Int J Biochem Cell Biol.

[84] Patzewitz EM, Guttery DS, Poulin B, Ramakrishnan C, Ferguson DJ, Wall RJ (2013). An ancient protein phosphatase, SHLP1, is critical to microneme development in Plasmodium ookinetes and parasite transmission. Cell Rep.

[85] Phillips MA, Lotharius J, Marsh K, White J, Dayan A, White KL (2015). A long-duration dihydroorotate dehydrogenase inhibitor (DSM265) for prevention and treatment of malaria. Sci Transl Med.

[86] Phillips MA, Rathod PK, Rueckle T, Matthews D, Burrows JN, Charman SA, Chackalamannil S, Rotella D, Ward SE (2017). Medicinal chemistry case history: discovery of the Dihydroorate dehydrogenase inhibitor DSM265 as an antimalarial drug candidate. Comprehensive medicinal chemistry III.

[87] Phyo AP, Jittamala P, Nosten FH, Pukrittayakamee S, Imwong M, White NJ (2016). Antimalarial activity of Artefenomel (OZ439), a novel synthetic antimalarial endoperoxide, in patients with Plasmodium falciparum and Plasmodium vivax malaria: an open-label phase 2 trial. Lancet Infect Dis.

[88] Phyo AP, Nkhoma S, Stepniewska K, Ashley EA, Nair S, McGready R (2012). Emergence of Artemisinin-resistant malaria on the western border of Thailand: a longitudinal study. Lancet.

[89] Pierrot C, Lafitte S, Dive D, Fraisse L, Brocard J, Khalife J (2005). Analysis of immune response patterns in naı¨ve and Plasmodium berghei-infected young rats following a ferroquine treatment. Int J Parasitol.

[90] Pinedo-Cancino V, Arista KM, Valle-Campos A, Saavedra-Langer R, Roca C, Ramos-Rincón JM (2022). Hematological profiles of malaria-infected patients in an endemic area of Peru. Rev Peru Med Exp Salud Publica.

[91] Pulcini S, Staines HM, Pittman JK, Slavic K, Doerig C, Halbert J (2013). Expression in yeast links field polymorphisms in PfATP6 to in vitro Artemisinin resistance and identifies new inhibitor classes. J Infect Dis.

[92] Puri SK, Dutta GP (2003). Blood schizontocidal activity of WR 238605 (Tafenoquine) against Plasmodium cynomolgi and Plasmodium fragile infections in rhesus monkeys. Acta Trop.

[93] Robert A, Coppel Y, Meunier B (2002). NMR characterisation of covalent adducts obtained by alkylation of heme with the antimalarial drug Artemisinin. Inorg Chim Acta.

[94] Rottmann M, McNamara C, Yeung BKS, Lee MCS, Zou B, Russell B (2010). A Spiroindolones, a new and potent chemotype for the treatment of malaria. Science.

[95] Saifi A, Beg T, Harrath AHA, Altayalan FSH, Quraishy SA (2013). Antimalarial drugs: mode of action and status of resistance. Afr J Pharm Pharmacol.

[96] Sato S (2021). Plasmodium-a brief introduction to the parasites causing human malaria and their basic biology. J Physiol Anthropol.

[97] Schmitt EK, Ndayisaba G, Yeka A, Asante KP, Grobusch MP, Karita E (2022). Efficacy of Cipargamin (KAE609) in a randomized, Phase II dose-escalation study in adults in sub-Saharan Africa with uncomplicated Plasmodium falciparum malaria. Clin Infect Dis.

[98] Schrader FC, Barho M, Steiner I, Ortmann R, Schlitzer M (2012). The antimalarial pipeline – an update. Int J Med Microbiol.

[99] Siddiqui JA, Aamar H, Siddiqui A, Essar MY, Khalid MA, Mousavi SH (2022). Malaria in Afghanistan: challenges, efforts and recommendations. Ann Med Surg.

[100] Sinden RE, Talman A, Marques SR, Wass MN, Sternberg MJE (2010). The flagellum in malaria parasites. Curr Opin Microbiol.

[101] Singh V, Kumar A (2015). The integral plasmodium life cycle phenomenon: gametocyte genes. Bacteriol Parasitol.

[102] Sinxadi P, Donini C, Johnstone H, Langdon G, Wiesner L, Allen E (2020). Safety, tolerability, pharmacokinetics, and antimalarial activity of the novel plasmodium phosphatidylinositol 4-kinase inhibitor MMV390048 in healthy volunteers. Antimicrob Agents Chemother.

[103] Solomon L, Okere HC, Daminabo V (2014). Understanding human malaria: further review on the literature, pathogenesis and disease control. Rep Opin.

[104] Sonopo MS, Pillay A, Chibale K, Marjanovic-Painter B, Donini C, Zeevaart JR (2016). Carbon14 radiolabeling and tissue distribution evaluation of MMV390048. J Label Compd Radiopharm.

[105] Stone W, Mahamar A, Smit MJ, Sanogo K, Sinaba Y, Niambele SM (2022). Single low-dose Tafenoquine combined with dihydroArtemisinin–piperaquine to reduce Plasmodium falciparum transmission in Ouelessebougou, Mali: a phase 2, single-blind, randomised clinical trial, Lancet Microbe. Lancet Microbe.

[106] Suh J, Kim JH, Kim JD, Kim C, Choi JY, Lee J (2022). Cost-benefit analysis of Tafenoquine for radical cure of Plasmodium vivax Malaria in Korea. J Korean Med Sci.

[107] Susan A, Charman SA, Barnes SA, Bathurst IC, Brun R, Campbell M (2011). Synthetic ozonide drug candidate OZ439 offers new hope for a single-dose cure of uncomplicated malaria. Proc Natl Acad Sci U S A.

[108] Tisnerat C, Dassonville-Klimpt A, Gosselet F, Sonnet P (2022). Antimalarial drug discovery: from quinine to the most recent promising clinical drug candidates. Curr Med Chem.

[109] Trouiller P, Olliaro P, Torreele E, Orbinski J, Laing R, Ford N (2002). Drug development for neglected diseases: a deficient market and a public-health policy failure. Lancet.

[110] Uneke CJ (2007). Impact of placental Plasmodium falciparum malaria on pregnancy and perinatal outcome in sub-Saharan Africa: I: introduction to placental malaria. Yale J Biol Med.

[111] Walsh DS, Wilairatana P, Tang DB, Heppner DG, Brewer TG, Krudsood S (2004). Randomized trial of 3dose regimens of Tafenoquine (WR238605) versus low dose primaquine for preventing plasmodium vivax malaria relapse. Clin Infect Dis.

[112] Webster R, Mitchell H, Peters JM, Heunis J, O’Neill B, Gower J (2023). Transmission blocking activity of low-dose tafenoquine in healthy volunteers experimentally infected with plasmodium falciparum. Clin Infect Dis.

[113] White NJ, Duong TT, Uthaisin C, Nosten F, Phyo AP, Hanboonkunupakarn B (2016). Antimalarial activity of KAF156 in falciparum and vivax malaria. N Engl J Med.

[114] White NJ, Pukrittayakamee ST, Phyo AP, Rueangweerayut R, Nosten F, Jittamala P (2014). Spiroindolone KAE609 for falciparum and vivax malaria. N Engl J Med.

[115] WHO (2019). World Health Organization malaria policy advisory committee (MPAC) meeting, October 2019: meeting report (No. WHO/CDS/GMP/2019.12).

[116] WHO, World Health Organization (2022). WHO guidelines for malaria, 3 June 2022 (No. WHO/UCN/GMP/ 2022.01 Rev. 2).

[117] Wicha SG, Walz A, Cherkaoui-Rbati MH, Bundgaard N, Kuritz K, Gumpp C (2022). New in vitro interaction-parasite reduction ratio assay for early derisk in clinical development of antimalarial combinations. Antimicrob Agents Chemother.

[118] Wu RL, Idris AH, Berkowitz NM, Happe M, Gaudinski MR, Buettner C (2022). Low-dose subcutaneous or intravenous monoclonal antibody to prevent malaria. N Engl J Med.

[119] Yeung BKS, Zou B, Rottmann M, Lakshminarayana SB, Ang SH, Leong SY (2010). Spirotetrahydro β-carbolines (spiroindolones): A new class of potent and orally efficacious compounds for the treatment of malaria. J Med Chem.

[120] Yipsirimetee A, Chiewpoo P, Tripura R, Lek D, Day NPJ, Dondorp AM (2022). Assessment in vitro of the antimalarial and transmission-blocking activities of Cipargamin and Ganaplacide in Artemisinin-resistant Plasmodium falciparum. Antimicrob Agents Chemother.

[121] Zhang R, Suwanarusk R, Malleret B, Cooke BM, Nosten F, Lau YL (2016). A basis for rapid clearance of circulating ring-stage malaria parasites by the spiroindolone KAE609. J Infect Dis.

